# Mesenchymal Stromal Cell-Derived Extracellular Vesicles Restore Thymic Architecture and T Cell Function Disrupted by Neonatal Hyperoxia

**DOI:** 10.3389/fimmu.2021.640595

**Published:** 2021-04-15

**Authors:** Monica Reis, Gareth R. Willis, Angeles Fernandez-Gonzalez, Vincent Yeung, Elizabeth Taglauer, Margaret Magaletta, Teagan Parsons, Alan Derr, Xianlan Liu, Rene Maehr, Stella Kourembanas, S. Alex Mitsialis

**Affiliations:** ^1^ Division of Newborn Medicine & Department of Pediatrics, Boston Children’s Hospital, Boston, MA, United States; ^2^ Department of Pediatrics, Harvard Medical School, Boston, MA, United States; ^3^ Program in Molecular Medicine, Diabetes Center of Excellence, University of Massachusetts Medical School, Worcester, MA, United States

**Keywords:** mesechymal stromal cell, extracellular vesicle (EV), neonatal immune system, hyperoxia (oxygen), thymus

## Abstract

Treating premature infants with high oxygen is a routine intervention in the context of neonatal intensive care. Unfortunately, the increase in survival rates is associated with various detrimental sequalae of hyperoxia exposure, most notably bronchopulmonary dysplasia (BPD), a disease of disrupted lung development. The effects of high oxygen exposure on other developing organs of the infant, as well as the possible impact such disrupted development may have on later life remain poorly understood. Using a neonatal mouse model to investigate the effects of hyperoxia on the immature immune system we observed a dramatic involution of the thymic medulla, and this lesion was associated with disrupted FoxP3^+^ regulatory T cell generation and T cell autoreactivity. Significantly, administration of mesenchymal stromal cell-derived extracellular vesicles (MEx) restored thymic medullary architecture and physiological thymocyte profiles. Using single cell transcriptomics, we further demonstrated preferential impact of MEx treatment on the thymic medullary antigen presentation axis, as evidenced by enrichment of antigen presentation and antioxidative-stress related genes in dendritic cells (DCs) and medullary epithelial cells (mTECs). Our study demonstrates that MEx treatment represents a promising restorative therapeutic approach for oxygen-induced thymic injury, thus promoting normal development of both central tolerance and adaptive immunity.

## Introduction

The effect of high oxygen exposure during the neonatal period on the developing organs outside the cardio-respiratory compartment is incompletely characterized. The most studied pathology associated with neonatal exposure to hyperoxia is bronchopulmonary dysplasia (BPD), a multifactorial chronic lung disorder increasingly recognized as a systemic disease with multiorgan involvement in addition to lung injury. Given the role of inflammation in the pathogenesis of BPD (1, 2), it is important to understand the effects of neonatal exposure to high oxygen on the neonatal immune system and the development of adaptive immunity, which occurs more robustly during the late embryonic and perinatal period in the thymus gland (3, 4).

The thymus houses a complex 3D network of cortical and medullary stromal and non-stromal cells that control the development of committed bone-marrow derived T cell precursors to mature naïve T cells and FoxP3^+^ regulatory T cells (5, 6). Paradoxically, despite the crucial role in the generation of naïve T cells and a plethora of organ-specific regulatory T cells, the thymus is the first organ of the body exhibiting age-associated degeneration resulting in a concomitant reduction in the T cell output. This degeneration is linked with cellular senescence as a response to stress induced by various stimuli, of which oxidative stress is one of the most damaging agents (7). A variety of animal studies have linked increased oxidant stress with a reduction in thymic size, whereby exposure to various stimuli inducing production of reactive oxygen species (ROS) resulted in increased cellular senescence and thymic involution (8–10). Nonetheless, the effect of neonatal exposure to high oxygen concentrations in the thymic morphology and function is still not well defined. In a baboon BPD model, investigations into thymic involution provided evidence that thymic architecture and T cell function are altered leading to autoreactivity and immunodeficiency (11). Further mouse studies of neonatal hyperoxia-induced lung injury demonstrated a dysregulation of thymopoiesis and altered adaptive immune responses which persisted into adulthood (12, 13). These data suggest that hyperoxia-induced thymic atrophy during the neonatal period results in long-term complications with an autoimmune component. Nonetheless, the consequences of hyperoxia-induced disruption of T cell immunity remains elusive.

Work from our group and others have shown mesenchymal stromal cell (MSC)-based therapies to have significant protective effects in experimental models of BPD (14, 15), also summarized by Augustine et al. (16). In fact, MSC therapies have been investigated in various preclinical models and clinical trials (17–19). It was recently revealed that the main vector in MSC-derived therapeutic effects on lung disease is comprised by extracellular vesicles (EVs) (20, 21) and, in particular, the smaller subpopulation (<150 nm in diameter) which includes exosomes, the EVs generated *via* the endocytic pathway (22). We and others have shown that early administration of purified human MSC-small extracellular vesicles (MEx), improves core histological and functional features of BPD (23–25). Our group has further demonstrated that MEx ameliorate lung architecture and function *via* the modulation of pulmonary macrophage phenotypes (23). Nonetheless, the mechanisms of action of MEx are incompletely understood and it is likely that their immune-modulatory properties are based on a systemic interaction with the target immune cells (26).

Thus, on the basis of this evidence, we sought to investigate the effects of neonatal exposure to hyperoxia on thymic morphology and T cell development and assess the modulatory effect of MEx in the architecture and function of this organ. Here, we showed that a single dose of MEx administration restored thymic medullary architecture and function disrupted by exposure to neonatal hyperoxia. We further show that MEx restored thymocyte counts and the development of FoxP3^+^ regulatory T cells and prevented T cell autoreactivity. Using single cell transcriptomics, we demonstrated that MEx treatment majorly modulates the transcriptome of thymic medullary cells responsible for antigen presentation, such as dendritic cells (DCs) and medullary thymic epithelial cells (mTECs). In response to MEx, these cells manifested an enrichment in the expression of genes related to maturation and antigen presentation and genes pivotal in cellular protection against oxidative stress-induced injury.

## Materials and Methods

### Cell Isolation and Culture

Human umbilical cord Wharton’s Jelly-derived mesenchymal stromal cells (WJ-MSCs) were isolated using a modification of the tissue explant method (27). Briefly, the umbilical cord was rinsed twice with Dulbecco’s phosphate-buffered saline (dPBS, Invitrogen), and cut longitudinally with removal of arteries and veins. The soft gel tissues were dissected into ~3-6 mm^2^ pieces and individually placed on 193 mm^2^ tissue culture dishes (24-well plates, Sigma) containing α-Modified Eagle Medium (αMEM, Invitrogen) supplemented with 20% fetal bovine serum (FBS, Invitrogen), 2 mM L-glutamine (Cassionlabs) and 1% penicillin/streptomycin (Gibco) and incubated at 37°C in a humidified atmosphere of 5% CO_2_ for 12 days. After regular addition of complete αMEM, the umbilical cord explants were carefully harvested, plates were washed 3 times with media and the plastic adherent cell colonies were detached with 0.1% trypsin-EDTA and seeded into 152 cm^2^ tissue culture dishes (Sigma) for further expansion. Cell counts and viability were assessed using trypan blue exclusion method (1:1 v/v) and analysed using a Cellometer Auto T4 (Nexcelom Biosciences). At passage 4, WJ-MSCs were seeded into 10-stack Corning CellSTACK^®^ cell culture plates (surface area: 6360 cm^2^, Sigma) for conditioning and exosome harvesting. WJ-MSCs were obtained from several umbilical cords and passage 3 cells were characterized for their surface protein phenotype, morphology and *in vitro* differentiation capacity as described by the ISCT guidelines (28). For primary outcomes, exosomes derived from human foreskin (dermal) fibroblast cells (HDFs), were used as biological controls. The establishment of HDF cell cultures was performed as described elsewhere (23) and HDF cultures and expansion were maintained similarly to WJ-MSCs.

### Isolation of WJ-MSC Derived EVs

**Figure 1:**
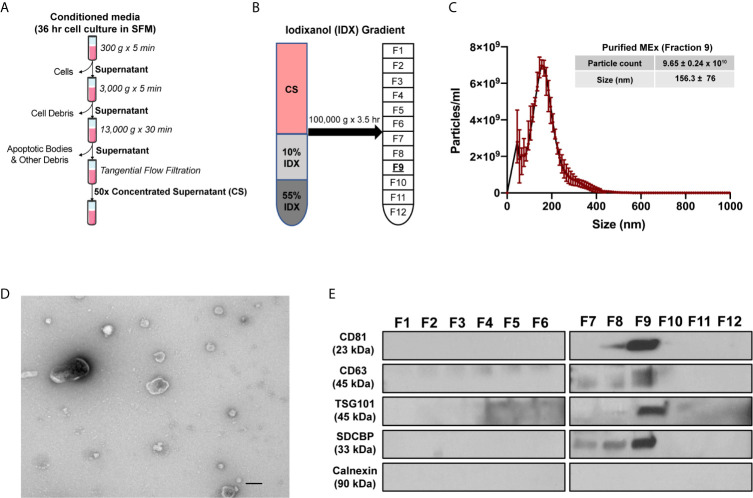
Purification and characterization of MEx. **(A)** To isolate MEx, the conditioned media was subjected to successive differential centrifugation followed by tangential flow filtration. **(B)** The concentrated supernatant (CS) was floated on an iodixanol (IDX) cushion gradient to purify and isolate MEx (fraction 9, density ~1.18g/ml). **(C)** Nanoparticle tracking analysis was utilized to assess MEx concentration and average size. The graph shows a representative size distribution particle count. Mean ± SD of size (nm) and particle counts are shown in the table. **(D)** Transmission electron microscopy image demonstrating heterogenous vesicle morphology (high magnification, 30,000x; scale bars = 100 nm). **(E)** Western blot analysis of the IDX fractions (1–12) using antibodies to proteins characteristic of exosome composition and non-exosomal protein marker. Equivalent volume of each fraction was loaded per lane and representative images are shown.

WJ-MSC derived EVs (MEx) were isolated as described previously (23). Briefly, confluent cells were incubated in serum-free media (SFM) for 36 hours. Cell culture media was then subjected to differential centrifugation at 300 x g for 10 minutes (to remove cells in suspension), followed by 3,000 x g for 10 minutes and 13,000 x g for 30 minutes for clearance of cell debris and large apoptotic bodies in suspension (**Figure 1A**). The resulting supernatant was then concentrated 50-fold by tangential flow filtration (TFF) using a modified polyethersulfone (mPES) hollow fibre with a 300 kDa MW cutoff (Spectrum Labs). Exosomes were further purified using an OptiPrep™ (Iodixanol; IDX) cushion density flotation. The IDX gradient was prepared by floating 3 mL of 10% w/v IDX solution containing 150 mM NaCl and 25 mM Tris : HCl (pH = 7.4) over 3 mL of 55% w/v IDX solution. The concentrated supernatant (6 mL) was then floated on top of the IDX cushion and ultracentrifuged for 3.5 hours at 100,000 x g at 4°C using a SW 40 Ti rotor (Beckman Coulter) (**Figure 1B**). Twelve 1 mL fractions were collected from the top of the gradient for immediate characterization or frozen (-1 °C/min) and kept at -80°C. HDF exosomes (FEx) were isolated from concentrated culture media similarly to MEx and used when appropriate as biological controls.

### Characterization of WJ-MSC Derived EVs

MEx characterization was performed following the standards recommended by the International Society for Extracellular Vesicles (29). For morphological assessment of the isolated vesicles, an aliquot of 25-10 μls of MEx suspension was adsorbed for 15 seconds onto a formvar/carbon coated grid (Electron Microscopy Sciences). Excess liquid was removed with a Whatman Grade 1 filter paper (Sigma) and adsorbed EVs were stained for 15 seconds with 2% uranyl acetate. EVs were assessed on a JEOL 1200EX transmission electron microscope (TEM) and images were acquired using an AMT 2k CCD Camera.

The concentration and size distribution of isolated EVs were measured by nanoparticle tracking analysis (NTA) using a NanoSight LM10 instrument (Malvern instruments). Samples were loaded using the NanoSight syringe pump and recorded under controlled flow. The camera shutter speed was fixed at 30.1 ms and camera gain to 500. The camera sensitivity and detection threshold were set for (11–14) and (4–6), respectively. For each sample, three videos of 60 seconds were recorded using the NTA software (version 3.0), and individual samples were run in triplicate. When appropriate, MEx samples were diluted in vesicle-free dPBS.

Assessment of protein content of specific EV markers was assessed by immunoblotting. In brief, proteins in EVs preparations were denatured, separated on a 4-20% polyacrylamide gel (BioRad), electro-blotted onto 0.45 μm PVFD membranes (Millipore) and probed with primary antibodies followed by incubation with horseradish peroxidase (HRP)-coupled secondary antibody. The primary antibodies used were mouse polyclonal anti-CD81 (1:200), anti-CD63 (1:200), anti-syntenin-1 (SDCBP, 1:100), anti-calnexin (1:200) all from Santa Cruz Biotechnology, and a rabbit polyclonal anti-Tsg101 (1:200) from Abcam.

### Animal Model and Experimental Design

**Figure 2:**
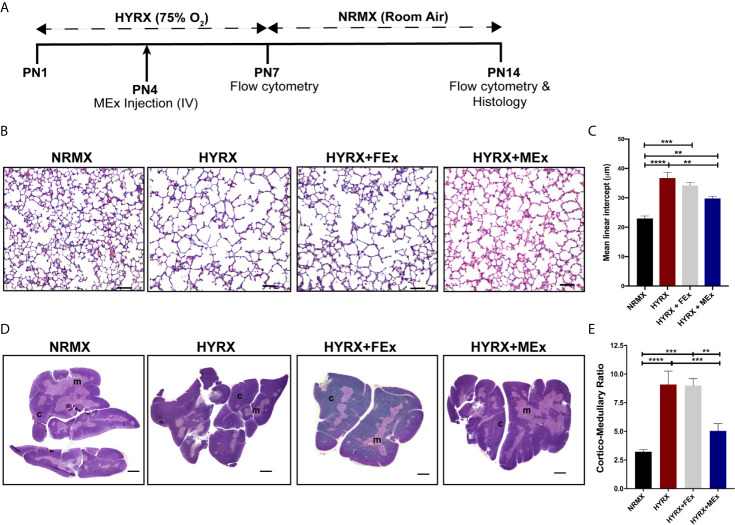
MEx restores thymic morphology after HYRX-induced injury. **(A)** Newborn mice (FVB strain) were exposed to hyperoxia (HYRX; 75% O2) for 7 days. HYRX-mice were compared with mice that were maintained in room air (NRMX). MEx treatments were administered intravenously (IV) at Postnatal day (PN) 4. Morphological outcomes were assessed at PN14. HYRX-exposed mice were compared with mice that remained at NRMX conditions for the duration of the study and human dermal fibroblast EV (FEx)-treated mice were used as biological controls. **(B)** HYRX-control and FEx treated mice presented with significant alveolar simplification compared to NRMX group. HYRX-mice that received a single dose of MEx had a significantly improved lung alveolarization compared to both HYRX- and FEx-controls. Images taken at 100x magnification and scale bars = 100 µm. **(C)** Quantification of mean linear intercept as a surrogate of average air space diameter. **(D)** Harvested thymi sections were stained for hematoxylin and eosin which evidences the cortex (dark purple areas) and the medulla (light purple areas) of the thymus. Images were taken at 40x magnification. Scale bars = 1000 µm. **(E)** Quantification of thymic cortico-medullary ratios in NRMX, HYRX, HYRX + FEx and HYRX + MEx groups. All data represent mean ± SEM n = 8-15 for each group (data pooled from at least two independent experiments). **P < 0.01, ***P < 0.001 and ****P < 0.0001.

The neonatal hyperoxia (HYRX)-induced BPD model used in this study has been extensively described and characterized in previous publications (14, 15, 23). All mice used in this study were produced in our specific pathogen-free facilities at Boston Children’s Hospital, and all experiments were conducted under protocols approved by the Boston Children’s Hospital Animal Care and Use Committee. In brief, time dated FVB pregnant mice were housed in separate ventilated cages under sterile conditions and pregnancy was closely monitored for delivery at around E21. Once the pups were delivered, the different litters were pooled and evenly split into different cages with a dam. The newborn mice were then exposed to hyperoxia, i.e., 75% O_2_, from postnatal day 1 (PN1) to postnatal day 7 (PN7) in a plexiglass hyperoxia chamber where, ventilation was adjusted by an Oxycycler controller (Biospherix) to remove CO_2_ so that it did not exceed 5,000 ppm (0.5%) and ammonia was removed by ventilation and charcoal filtration through an air purifier. HYRX-exposed mice were compared to mice that remained in room air from birth (Normoxia, NRMX) and dams were rotated between NRMX and HYRX cages every 48 hours to prevent excessive O_2_ related toxicity to the adult mice. At PN7, hyperoxia-exposed mice were placed in room air for another 7 days. The thymi of mice from the different experimental groups were harvested at PN7 and PN14 for flow cytometric analysis, single cell RNA sequencing and histological assessment. Lungs and spleens were also harvested from PN14 mice for histology and flow cytometric analysis (**Figure 2A**).

### MEx Dosing

The MEx dose used in this study was based on previous studies published by our laboratory (20, 23). At PN4, EV preparations from WJ-MSCs and HDFs were injected intravenously (IV) *via* the superficial temporal vein. Preparations were diluted accordingly in dPBS to achieve a dose per pup corresponding to the product generated by 0.5 x 10^6^ cells.

### Histology and Immuno-Histochemistry/Fluorescence

Mice were anesthetized with 60 mg/kg of pentobarbital (intraperitoneal – IP) and perfused with PBS through the right ventricle at a constant pressure of 25 cmH_2_O. Thymi were collected onto a tube with 4% paraformaldehyde (PFA) and stored overnight at 4°C. For lung collection, the right lung was inflated to a fixed pressure of 15-50 cm H_2_O with 4% PFA *in situ* and stored as described for the thymus. Fixed tissues were then transferred to 75% ethanol (EtOH) before subsequent processing and paraffin embedding for sectioning. All tissues were processed at the Rodent Histopathology Core at Harvard Medical School or by Servicebio Inc. Two sections per tissue were stained with hematoxylin and eosin for analysis. Thymic cortico-medullary ratios were assessed by imaging whole thymus using a Nikon Eclipse 80i microscope (Nikon) and assessing cortical and medullary area using ImageJ v2.0 software (ImageJ). For assessment of lung histology, randomly selected areas (10-20 fields) were acquired and mean linear intercept (MLI) was measured as previously described (14, 23) using Metamorph software v.6.2r (Universal Imaging).

For immunofluorescence analysis, thymic tissue sections were de-paraffinized in xylene and rehydrated. Tissue slides were treated with a 10 mM sodium citrate buffer for 20 minutes for antigen retrieval and blocked with blocking solution (1X PBS with 0.4% of 20% Triton X-100, 1% BSA and 4% of goat serum) for 30 minutes. Tissue slides were then incubated with monoclonal anti-mouse antibodies, (AIRE and CD11c at a dilution of 1:400, Thermo Fisher Scientific, and FoxP3 and CD4 at a dilution of 1:1000 and 1:2000, respectively, Cell Signalling Technologies and Servicebio Inc) followed by incubation with secondary antibodies at a dilution of 1:200 (Rhodamine red™-X goat anti-Rabbit IgG and Alexa Fluor^®^ 488-goat anti-Armenian Hamster IgG, Molecular probes and Jackson ImmunoResearch, respectively) and 4’,6’-diamidino-2-phenylindole (DAPI, 1:1000, Thermo Fisher Scientific). Tissue slides were imaged as described above and quantification of Aire, CD11c and FoxP3 staining was calculated using ImageJ software. Apoptosis in thymic tissue sections was investigated by immunohistochemistry using Terminal deoxynucleotidyl transferase dUTP nick end (TUNEL) using the TACS^®^ TdT kit with colorimetric substrate diaminobenzidine (DAB, R&D Systems), and performed as described by the manufacturer’s protocol.

### Tissue Preparation of Cell Suspensions

Mice were harvested at PN7 and PN14. Thymus, lungs and spleen were harvested, minced, transferred to falcon tubes (Thermo Fisher Scientific) and processed in 5 ml of digestion media consisting of RPMI-1640 (Invitrogen), 1.6 mg/ml of Collagenase IV and 50 Unit/ml of DNase1, both from Worthington Biochemical Corp. Homogenized tissues were passed through a 40 µm cell strainer (Corning) to obtain a single cell suspension. The remaining red blood cells were lysed using red blood cell lysis buffer (Roche). The resulting single cell suspensions were washed with and resuspended in PBS containing 0.5% BSA and used for further experiments.

For single cell RNA sequencing of thymic cell suspensions, PN7 and PN14 thymi were dissociated with RPMI-1640 containing 0.5 U/ml of Liberase (Roche) and 100 µg/ml DNAse1 at 37°C for 25 minutes with mechanical dissociation by pipetting every 5 minutes. The remaining tissue pieces were passed through a 100 µm cell strainer and the dissociation media was neutralized with RPMI-1640 media supplemented with 10 mM HEPES (Gibco), 1% penicillin and streptomycin, 2 mM of L-glutamine and 10% FBS. Red blood cells were lysed, and single-cell suspensions were washed and resuspended as mentioned above. Cells were transferred into 5 ml polypropylene round-bottom tubes (Corning) and dead cells were detected by 7-aminoactinomycin D (7-AAD; 1:60, eBioscience) staining and assessed using a BD Accuri™ flow cytometer (BD Biosciences). For single cell RNA sequencing experiments, 3 thymi of each group (NRMX, HYRX and HYRX+MEx) were harvested and pooled.

### Flow Cytometry

Mouse thymic, splenic and lung T lymphocytes were stained using the following antibodies: Brilliant Violet 510 (BV510) anti-CD3e (145-C211) from BD Biosciences; Phycoerythrin : Cy-7 (PE-Cy7) anti-CD4 (GK1.5), Peridinin-chlorophyll-protein complex: Cy 5.5 conjugate (PerCP-Cy5.5) anti-CD8b (YTS156.7.7), Allophycocyanin (APC) fire 750 anti-CD45 (30-F11), Fluorescein Isothiocyanate Conjugate (FITC) anti-CD25 (PC61), Phycoerythrin (PE) anti-CD44 (IM7) and Brilliant Violet 421 (BV421) anti-FoxP3 (MF-14) from BioLegend. Human-derived WJ-MSCs were assessed for the expression of established MSC markers, using the following antibodies: APC anti-CD105 (43AE), FITC anti-CD90 (5E10), PE anti-CD14 (63D3), PE anti-CD19 (4G7) and PE anti-CD34 (561) from BioLegend; PE anti-CD73 (AD2), FITC anti-CD44 (G44-26), PE anti-CD146 (P1H12), FITC anti-HLA-DR (G46-6) and FITC anti-CD11b (M1/70) from BD Biosciences. Briefly, single cell suspensions were washed in flow cytometry and fluorescence-activated cell sorting (FACS) buffer consisting of 1X dPBS supplemented with 0.5% bovine serum albumin (BSA, Rockland) and surface stained for 20 minutes with fluorochrome-conjugated antibodies at room temperature, before washing and analysis or intracellular staining. Intracellular staining of FoxP3 was performed using the BD Biosciences Cytofix/Cytoperm™ kit as described by the manufacturer’s protocol. All data was acquired on a BD LSR Fortessa™ flow cytometer using BD FACSDiva software (BD Biosciences). Compensation was adjusted accordingly, fluorescence-minus-one was used as controls and cell populations were identified using sequential gating strategy (**Figures S1** and **S2**). When stated, cell quantification was performed using CountBright™ Absolute Counting Beads (ThermoFisher) as recommended by the manufacturer. All data were analysed using FlowJo software v10.5.0 (Tree Star).

### Assessment of Autoreactive T Lymphocytes

Assessment of autoreactive T lymphocytes was performed as previously described (11). To assess whether hyperoxia reprogrammed T lymphocytes into an autoreactive phenotype, newborn mice were exposed to hyperoxia for 7 days and MEx treatments were carried out at PN4 as described above. The mice were moved to normal air conditions at PN7 and kept alive until PN28, when thymus, spleen and lungs were harvested, and single cell suspensions were prepared as described above (**Figure 5A**). Lung stimulator cells were sub-lethally irradiated with 15 Gy, re-suspended in RF10 media, i.e., RPMI-1640 supplemented with 10% FBS and 1% Penicillin/streptomycin, and plated at a concentration of 2x10^5^ cells/well in a round bottom 96-well plate. The responder cells, i.e., thymocytes and splenocytes, were labelled with 0.5 µM Carboxyfluorescin succinimidyl ester (CFSE, BD Biosciences), re-suspended in RF10 and plated onto the wells of the 96-well plate containing lung cells at a concentration of 2x10^5^ cells/well. For negative controls, cells were cultured in RF10 alone and for positive controls, the cells were plated with mouse Dynabeads™ anti-CD3/anti-CD28 stimulator beads (Thermo Fisher Scientific) at a bead to cell ratio of 1:1 and 30 IU/ml of IL-2 (R&D Systems). Plates were incubated at 37°C in 5% CO_2_ for 5 days, after each they were harvested and analysed for CFSE dilution using flow cytometry. Gating of CD4 and CD8 cells was performed as previously described (**Figures S1** and **S2**).

### Single Cell RNA Sequencing of Thymi Using 10x Genomics Chromium

For single cell RNA sequencing (scRNA-seq), thymi from PN7 mice were harvested and and processed as previously described (30). Three thymi of each group, i.e., NRMX, HYRX and HYRX+MEx, were processed into single cell suspensions and pooled. Single cell suspensions were assessed for their viability and used for scRNA-seq using the 10x Genomics chromium platform. Live cells were loaded into a Chromium controller (10x Genomics, Inc.) and single-cell cDNA libraries were generated using v3 chemistry according to the manufacturer’s protocol (10x Genomics, Inc.) A total of 3 cDNA libraries (PN7: 1 NRMX, 1 HYRX and 1 HYRX+MEx) were multiplexed and sequenced on one lane of Illumina NextSeq High Output 75 cycle kit v2.5. A list of all reagents used is described in **Table S1**. For mapping, raw base call files obtained from sequencing were demultiplexed using the 10x Genomics Cell Ranger pipeline (v2.1.0) and aligned to the mouse mm10 transcriptome. Count matrices obtained with the 10x Genomics Cell ranger pipeline for the three tested conditions were combined and further analysis, including quality filtering, the identification of highly variable genes, dimensionality reduction, standard unsupervised clustering algorithms and the discovery of differentially expressed genes was performed using the Seurat v3 R package (31). Only confidently mapped, non-PCR duplicates with valid barcodes and unique molecular identifiers were used to generate the gene-barcode matrix that contained the cell numbers shown in **Table S2**. Briefly, cell type identification and clustering were performed in two different stages. Firstly, the data was normalized and scaled, and the 2000 most variable genes were identified followed by a combination of Principle component analysis (PCA) and uniform manifold approximation and projection (UMAP) for dimensionality reduction to a two-dimensional map. Clusters were then identified based on the most significant marker genes for each cluster. Based on these results, the initial analysis identified large clusters of cells composed of empty droplets (cell suspension buffer containing RNA from lysed cells) and T cells. Since we were primarily focused on the non-T cell fraction of the data set, T cell clusters were removed before further analysis. Primary differential expression results for T cells and non-T cells are shown in **Tables S3** and **S4**. For the second stage of analysis on the already reduced data, we identified only the top 200 most variable genes for PCA, UMAP, clustering and cluster marker gene analysis. Using the marker genes, we identified three clusters of interest: Cluster 2 (*Siglech* dendritic cells), Cluster 5 (*Aire* medullary thymic epithelial cells) and Cluster 6 (*Xcr1* dendritic cells). Finally, we selected the cells from each test condition from each cluster of interest and used R Bioconductor package edgeR to identify genes that were significantly differentially expressed between the analyzed groups. Using Ingenuity Pathway Analysis (IPA), potential mRNA enriched canonical pathways and were explored. Genes for clusters 02, 06 and 05 that exhibited a False Discovery Rate < 0.05 for the pairwise comparisons HYRX vs NRMX and HYRX+MEx vs HYRX were uploaded onto IPA for core analysis. Afterwards, canonical pathway enrichment for the two pairwise comparisons for each analyzed cluster were compared using the comparison module in IPA. Only pathways showing a Z score >2 or <-2 in the comparison HYRX+MEx vs HYRX, were considered in which positive and negative Z scores represented activated and inhibited pathways, respectively. Gene lists for relevant pathways were downloaded from IPA for further analysis.

### Statistical Analysis

Unless otherwise stated, all statistical analyses were performed using GraphPad Prism v8.0 software (GraphPad Inc.). Data are presented as mean ± SEM and all *in vivo* experiments were repeated more than two times. Statistical significance, as indicated by asterisks, was determined by one-way ANOVA and P < 0.05 was considered significant (*P < 0.05; **P < 0.01; ***P < 0.001, ****, P<0.0001). 

## Results

### Isolation and Characterization of MEx

MSCs were obtained from umbilical cord Wharton’s Jelly (WJ-MSCs) by an adaptation of previously described methods (27) and characterized as recommended by the International Society of Cellular Therapies (ISCT) guidelines (28). WJ-MSCs conformed to the ISCT criteria and exhibited a spindle-shaped morphology and the capacity to differentiate into adipocytes and osteocytes. WJ-MSCs were >95% positive for the expression of CD73, CD90, CD104, CD44 and CD46, and negative (>98%) for the expression of hematopoietic lineage specific markers and HLA-DR (**Figure S3**). The conditioned media (CM) from WJ-MSCs were harvested after a 36hr incubation of confluent cells in serum-free media and floated on an iodixanol (IDX) cushion (**Figures 1A, B**). MEx were extracted in fraction 9 (F9, density of ~1.18g/ml) of the gradient and characterized in accordance with the 2018 Minimal Information for Studies of Extracellular Vesicles (MISEV) (29), as outlined by the International Society for Extracellular Vesicles (ISEV). Transmission Electron Microscopy (TEM) and Nanoparticle Tracking analysis (NTA) of F9 revealed a heterogeneous population of MEx exhibiting a biconcave morphology with minimal protein aggregate contaminants and mean size of 156.3 ± 76 nm in diameter at a concentration of 9.65 ± 0.24 x 10^10^ particles/ml (**Figures 1C, D**). Immunoblots of the 12 fractions extracted from the IDX cushion gradients revealed an enrichment of the EV-associated markers: CD81, CD63, TSG101 and syntenin-1 (SDCBP) in F9, with all fractions being negative for Calnexin, a protein present in the endoplasmic reticulum, indicating low cytoplasmic contamination levels (**Figures S4** and **1E**).

### A Bolus Dose of MEx Improves the Architecture of Neonatal HYRX-Induced Thymic Injury

Newborn mice were exposed to 75% O_2_ (hyperoxia, HYRX) from postnatal day (PN) 1 to PN7 and returned to room air from PN7 to PN14. Age-matched control litters were maintained at room air conditions (normoxia, NRMX) for the duration of the study. At PN4, the treatment groups received a bolus dose of MEx at a volume corresponding to vesicles secreted by 5x10^5^ WJ-MSCs (**Figure 2A**). In accordance with our previous studies (14, 15, 23), pups exposed to HYRX presented with histopathological findings consistent with alveolar simplification at PN14, which were ameliorated after MEx treatment (**Figures 2B, C**). At PN14, the HYRX group pups exhibited a strikingly reduced thymic medullary area which was reflected in increased cortico-medullary ratios compared to NRMX-controls (3.22 ± 0.19 vs. 9.09 ± 1.18, p < 0.0001, respectively). MEx treatment of HYRX-exposed mice restored thymic medullary areas as shown by a lower cortico-medullary ratio compared to the HYRX group (5.04 ± 0.63, p = 0.0003) (**Figures 2D, E**). Human dermal fibroblast-exosomes (FEx), which served as biological control, had no protective effect on amelioration of lung simplification and thymic architecture at PN14 and were therefore, not used in further experiments (**Figures 2B–E**).

### MEx Treatment Restores Thymocyte Counts and Promotes Their Maturation Into a CD4^+^ Phenotype

**Figure 3:**
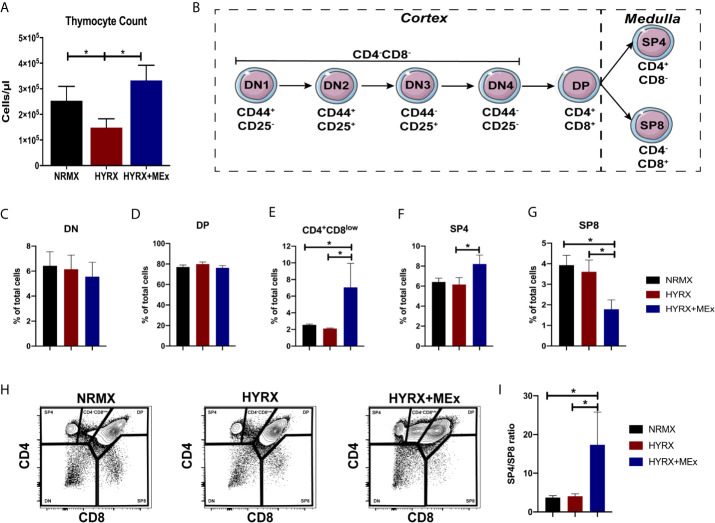
MEx restores thymocyte counts and promotes the differentiation of SP4 thymocytes. **(A)** Thymocyte counts from NRMX, HYRX and MEx treated groups was assessed at PN14 using CountBright™ absolute counting beads by flow cytometry. **(B)** Schematic of thymocyte phenotype development in the thymus. Frequencies of **(C)** DN, **(D)** DP, **(E)** CD4^+^CD8^low^ intermediate population, **(F)** SP4 and **(G)** SP8 thymocytes in thymic single cell suspensions harvested from NRMX, HYRX and HYRX + MEx mice at PN14. **(H)** Representative flow cytometry graphs showing the specification of the different thymocyte populations, i.e., DN, DP, SP4 and SP8, assessed by analyzing the expression of CD4 and CD8. **(I)** SP4/SP8 ratio of the three analyzed groups. Data derived from three independent experiments, N = 8-13. Data represents mean ± SEM and *P < 0.05.

The effect of neonatal oxygen exposure on thymic development is still not fully understood, but previous studies have demonstrated that thymi from HYRX-exposed animals presented with lower thymocyte counts (11–13). Thus, we investigated thymocyte counts in thymi from the NRMX, HYRX and HYRX+MEx groups. Compared to NRMX-control, the HYRX group exhibited a significant reduction in thymocyte counts at PN14 (2.53 ± 0.56 x 10^5^ vs. 1.48 ± 0.34 x 10^5^ cells/μl, p = 0.05 respectively). MEx treatment of HYRX-exposed mice restored thymocyte counts to levels akin to NRMX (3.32 ± 0.59 x 10^5^, p = 0.03) (**Figure 3A**). Thymocyte development is a microenvironment-controlled process that can be tracked in detail through a combination of surface markers. The earliest stages of thymocyte development (double negative, DN1-4) is characterized by the differential expression of CD44 and CD25. Upon TCR rearrangements, these cells specify into double positive (DP) CD4^+^CD8^+^ T cells which, by migrating into the thymic medulla, further differentiate into either single CD4^+^ (SP4) or CD8^+^ (SP8) (**Figure 3B**). Freshly isolated thymocytes from the three experimental groups were analyzed for their phenotypes at PN7 and PN14. Cells were labelled for the expression of CD44, CD25, CD4 and CD8 and gated as previously reported (32) and presented in **Figure S1A**. At PN7, no differences were detected in the frequencies of DN, DP, SP4 and SP8 across the three groups (**Figure S5**). At PN14, no differences were detected in the frequencies of the DN and DP stages across NRMX, HYRX and HYRX+MEx groups (**Figures S6**, **3C, D, H**). Interestingly, MEx-treated mice presented with elevated frequencies of the intermediary phenotype CD4^+^CD8^low^ compared to both NRMX and HYRX-groups (**Figures 3E, H**, 7.06 ± 2.89% vs 2.56 ± 0.10 and 2.11 ± 0.07, respectively, p = 0.04 and p = 0.03). HYRX-exposed mice that received MEx treatment showed an increased proportion of SP4 compared to HYRX controls (8.20 ± 0.89% vs. 6.14 ± 0.69% respectively, p = 0.05) but there were no significant differences in SP4 cells between NRMX and the other groups (p > 0.05). In contrast, the frequency of SP8 cells was lower in HYRX+MEx group compared to their HYRX or NRMX counterparts (1.78 ± 0.459% vs. 3.61 ± 0.57, p = 0.029 and 3.93 ± 0.48%, p = 0.012 respectively) (**Figures 3F-H** **)**, which translated into higher SP4/SP8 ratio in the thymi of HYRX+MEx mice compared to both HYRX and NRMX groups (17.36 ± 8.42 vs. 4.06 ± 0.634 and 3.75 ± 0.463, respectively, p < 0.05) (**Figure 3I**). These data indicate a preferential specification of SP4 thymocytes in the HYRX+MEx group.

### MEx Treatment Restores the Generation of Thymic Regulatory T Cells in Neonatal HYRX-Exposed Mice

**Figure 4:**
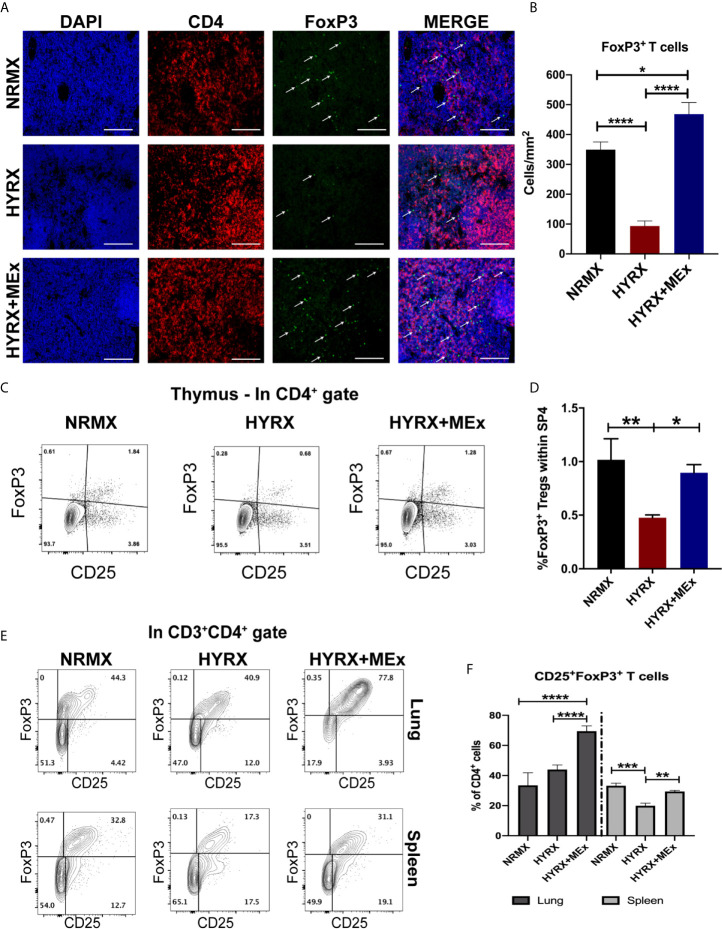
MEx treatment promotes a regulatory T cell phenotype at multiorgan level. **(A)** Representative images of PN14 thymus sections stained for DAPI (blue), CD4 (red) and FoxP3 (green) expression from the three experimental groups. Images were obtained at 200x magnification and scale bar = 50 µm. **(B)** Quantification of FoxP3 expressing cells in thymi of NRMX, HYRX and HYRX+MEx groups expressed as cells/mm^2^. Data represent means of 5 thymic sections per mice. **(C)** Representative and **(D)** Quantitative flow cytometry data of FoxP3 expression within the SP4 (CD4^+^CD8^-^)CD25^+^ thymocyte population of PN14 thymi harvested from the three experimental groups. **(E)** Representative flow cytometry graphs of FoxP3 expression within the CD3^+^CD4^+^ T cell population in cell suspensions obtained from lung (upper panel) and spleen (lower panel) in NRMX, HYRX and HYRX + MEx. **(F)** Frequencies of FoxP3 expressing cells within the CD4 population from lung (black bars) and spleen (grey bars) in the three experimental groups. Data obtained from at least two independent experiments representing mean ± SEM of N = 8-13 and *P < 0.05, **P < 0.01, ***P < 0.001, ****P < 0.0001.

To assess thymic regulatory T cell phenotypes in NRMX, HYRX and HYRX+MEx groups, PN14 thymi were harvested and assessed for the expression of FoxP3 by immunofluorescence and flow cytometry (using the gating strategy shown in **Figure S1B**). Compared to the NRMX group, HYRX-thymi exhibited a reduced proportion of CD4^+^FoxP3^+^ regulatory T cells (349.6 ± 25.51 vs. 93.63 ± 16.66 cells/mm^2^, respectively, p < 0.0001). In contrast, HYRX+MEx group presented a dramatically increased frequency of FoxP3 expressing T cells compared to the HYRX group (468.1 ± 38.91 cells/mm^2^ vs. 93.63 ± 16.66 cells/mm^2^, p < 0.0001) and a smaller, although significant increase above the NRMX group (468.1 ± 38.91 cells/mm^2^ vs. 349.6 ± 25.51, p < 0.05) (**Figures 4A, B**). Similar restoration of FoxP3 expressing T cells by MEx treatment was shown using a flow cytometric approach (**Figures 4C, D**). To further explore whether MEx influenced regulatory T cell phenotypes at a multi-organ level, spleen and lung cell suspensions derived from the three experimental groups at PN14 were analyzed for FoxP3 expression within the CD4^+^CD25^+^ T cell populations (gating strategy shown in **Figure S2**). Increased frequencies of CD4^+^CD25^+^FoxP3^+^ T cells were detected in HYRX+MEx compared to NRMX and HYRX groups in the lung (69.50 ± 3.55% vs. 33.47 ± 8.28% and 43.92 ± 3.03% of CD4 cells, respectively, p < 0.0001). In the spleen, the frequencies of CD4^+^CD25^+^FoxP3^+^ T cells were comparable in HYRX+MEx and NRMX-mice, however, the frequency of these cells in the HYRX+MEx group was greater than in HYRX-mice (29.43 ± 0.73% vs. 19.90 ± 1.74% of CD4 cells, p = 0.008) (**Figures 4E, F**). These data indicate that MEx treatment restores the levels of FoxP3^+^ T cells lost during HYRX exposure at a multi-organ level.

### HYRX-Induced Autoreactive T Cells Are Suppressed by MEx Administration

**Figure 5:**
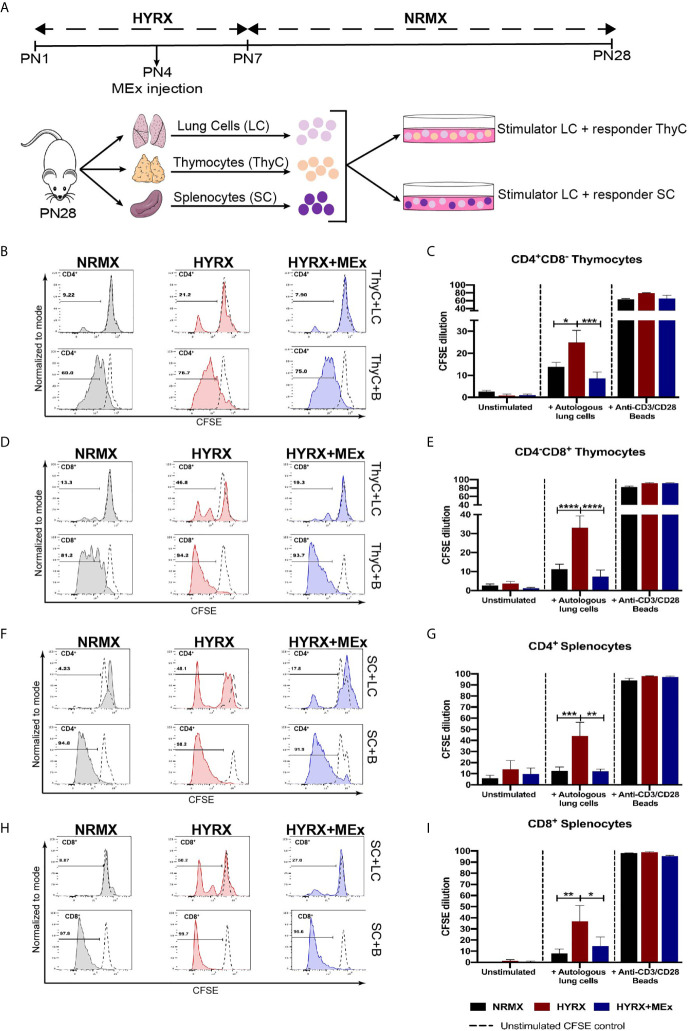
MEx treatment reduces hyperoxia-induced T cell autoreactivity. **(A)** Schematics showing the experimental layout to test autoreactivity after hyperoxia exposure. Newborn mice were exposed to HYRX as previously described for 7 days with MEx treatments being performed as PN4. Mice were then moved to normoxia conditions for a further period of 14 days. HYRX-mice were compared to mice kept in room air (NMRX) for the 28-day period. At PN28, lungs, spleens and thymi were harvested and single cell suspensions were prepared. Splenocytes (SC) or thymocytes (ThyC) were co-cultured at a 1:1 ratio with autologous lung cells (LC). Co-cultures were maintained, and T cell proliferation was analyzed as described in the methods section. Splenocytes and thymocytes cultured in media alone or in the presence of anti-CD3/CD28 stimulator beads were used as negative and positive controls respectively. Representative graphs and quantitative CFSE dilution of frequencies of CD4^+^CD8^-^ **(B, C)** and CD4^-^CD8^+^ **(D, E)** thymocytes from co-cultures with autologous LC (ThyC+LC; upper panels) and stimulator beads (ThyC+B; lower panels). Representative graphs and quantitative CFSE dilution frequencies of CD4^+^ **(F, G)** and CD8^+^ **(H, I)** splenocytes from co-cultures with autologous LCs (SC+LC; upper panels) and stimulator beads (SC+B; lower panels). Data derived from two independent experiments representing mean ± SEM of N = 6 and *P < 0.05, **P < 0.01, ***P < 0.001, ****P < 0.0001.

One of the main functions of T cell thymic selection is the identification and elimination of autoreactive T cells. A previous study has demonstrated increased T cell autoreactivity as a direct effect of exposure to HYRX during the neonatal period (11). As an approach to evaluate T cell autoreactivity, co-cultures of single cell suspensions of CFSE-labelled splenocytes and thymocytes with sub-lethally irradiated lung cells were performed as described in the methods section and shown in **Figure 5A**. After a 5-day co-culture, cells were harvested and analyzed for CFSE dilution using flow cytometry and the gating strategy described in **Figure S2D**. Thymocytes and splenocytes from the three different experimental groups cultured in media alone (unstimulated) and in the presence of anti-CD3/CD28 activation beads were used as negative and positive controls, respectively. Both thymocytes and splenocytes showed negligible proliferation of the CD4^+^ and CD8^+^ T cells in the presence of media alone and high-proliferative capacity (>60% of CFSE dilution for both CD4 and CD8 cell populations) in the presence of anti-CD3/CD28 beads (**Figures 5B–I**). Overall, both CD4^+^CD8^-^ and CD8^+^CD4^-^ thymocytes obtained from the HYRX group manifested increased proliferation, as shown by greater proportion of CFSE dilution, in response to autologous lung cells. In HYRX-control, CD4^+^CD8^-^ thymocytes exhibited a proliferation rate of 24.92 ± 5.51% compared to 13.89 ± 2.02% and 8.66 ± 2.79% in the NRMX and HYRX+MEx groups, respectively (p < 0.05) (**Figures 5B, C**). Similarly, CD8^+^CD4^-^ thymocytes obtained from HYRX-mice co-cultured with autologous lung cells, manifested increased proliferation compared to NRMX and HYRX+MEx group (33.04 ± 6.18% vs. 11.20 ± 2.71 and 7.27 ± 3.52%, respectively, p < 0.0001) (**Figures 5D, E**). Equivalent responses to autologous lung cells were demonstrated by both CD4^+^ and CD8^+^ splenocytes. Compared to NRMX and HYRX+MEx CD4 splenocytes, which exhibited proliferation rates of 12.65 ± 3.38% and 12.33 ± 1.87%, respectively, HYRX-CD4 splenocytes proliferated at an increased frequency (44.12 ± 12.13%, p < 0.05) (**Figures 5F, G**). Splenocytes of the CD8 lineage from HYRX group proliferated at a rate of 36.89 ± 14.14% which was greater than the proliferation rates of CD8^+^ splenocytes derived from either NRMX or HYRX+MEx groups (8.01 ± 3.94 and 14.46 ± 8.2, respectively, p < 0.05) (**Figures 5H, I**). In all analyzed comparisons, no differences were detected between HYRX+MEx group and NRMX-controls.

### Medullary Thymic Epithelial Cell Aire and DC-Specific CD11c Expression Is Impaired by HPRX and Restored by MEx Treatment

**Figure 6:**
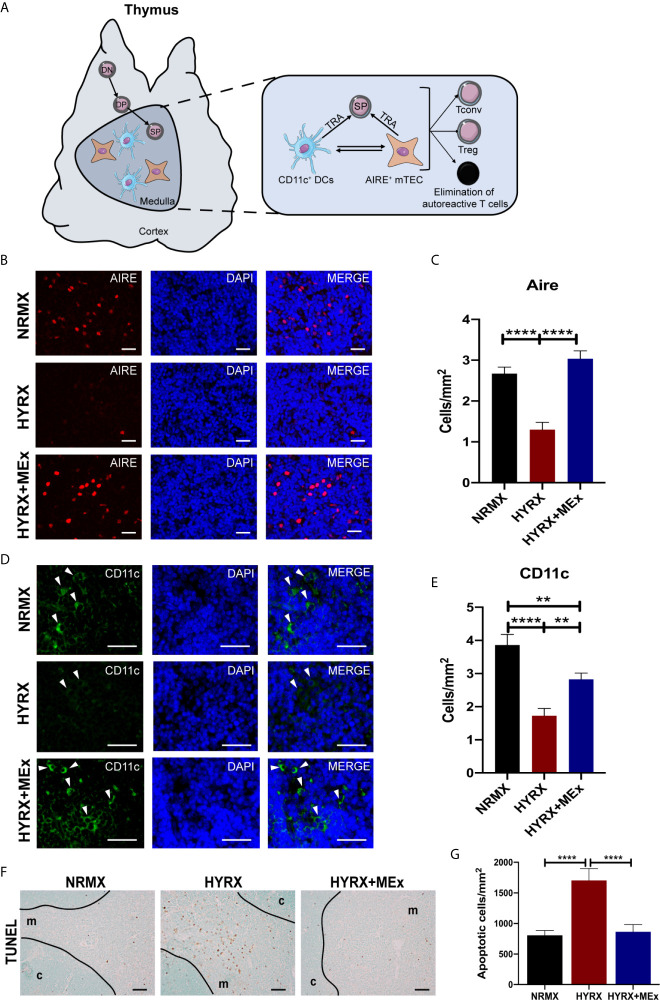
MEx restores Aire and CD11c expression by thymic medullary antigen presenting cells and blunts HYRX-induced apoptosis. **(A)** Schematics showing the role of thymic medulla in thymocyte development and establishment of immune tolerance by elimination of autoreactive T cells and generation of regulatory T cells (Treg) and conventional T cells (Tconv) in a process regulated by tissue restricted antigen (TRA) expressing Aire^+^ medullary thymic epithelial cells (mTECs) and dendritic cells (DCs, CD11c^+^ cells). **(B)** Representative images of PN14 thymus sections stained for Aire (red) and DAPI (blue) from NRMX, HYRX and HYRX+MEx groups. Images were obtained at a 400x magnification and scale bars = 50 µm. **(C)** Quantification of Aire^+^ cells (cells/mm^2^) in the three experimental groups. **(D)** Representative images of PN14 thymus sections stained for CD11c (green) and DAPI (flue) from the three analyzed experimental groups. Images were obtained at 600x magnification and scale bars = 50 µm. **(E)** Quantification of CD11c^+^ cells (cells/mm^2^) in the thymic medulla in NRMX, HYRX and HYRX+MEx. **(F)** Representative images of TUNEL staining in NRMX, HYRX and HYRX+MEx. Cortical (c) and medullary (m) boundaries delineated with a black line. Images were taken at 200x magnification and scale bars = 50 µm. **(G)** Quantification of TUNEL positive cells in thymi from NRMX, HYRX and MEx treated mice. Data from at least three independent experiments representing mean ± SEM of N = 8-15. **P < 0.01, ****P < 0.0001.

Results herein demonstrated a disruptive effect of exposure to neonatal HYRX on the thymic medulla, which is restored upon administration of a single dose of MEx. The thymic medulla is essential for the establishment of central tolerance. After thymocytes undergo positive selection in the thymic cortex, they differentiate into CD4^+^ or CD8^+^ single positive (SP) cells that may recognize self-peptide/MHC complexes (5, 6). In the thymic medulla a complex network of epithelial cells (mTECs) expressing autoimmune regulator transcription factor (Aire) interact with medullary dendritic cells (DCs) to present MHC bound tissue restricted antigens (TRAs) to the developing SP cells. These cells orchestrate the elimination of autoreactive T cells by apoptosis and the generation of regulatory T cells (Tregs) and conventional T cells (Tconv) (**Figure 6A**). We analyzed the effect of HYRX-exposure on these thymic medullary cells by quantifying Aire^+^ mTECs and CD11c^+^ DCs by immunofluorescence. Thymi harvested from the HYRX group presented with reduced numbers of Aire^+^ mTECs compared to NRMX-controls (1.303 ± 0.1760 vs 2.671 ± 0.1619 cells/mm^2^ of medulla, respectively, p < 0.0001), which was increased by MEx treatment of HYRX-exposed mice (3.035 ± 0.1945 cells/mm^2^, p < 0.0001) (**Figures 6B, C**). Similarly, numbers of CD11c^+^ cells were reduced in the thymi of HYRX-exposed mice compared to the NRMX group, indicative of a loss of medullary DCs (1.725 ± 0.2215 vs 3.859 ± 0.3240 cells/mm^2^, respectively, p < 0.0001). MEx treatment of HYRX-exposed mice induced an increase of CD11c^+^ cells in the thymic medulla compared to HYRX-group (2.826 ± 0.1899 cells/mm^2^, p = 0.0020) (**Figures 6D, E**). Additionally, TUNEL staining showed a significant increase in the percentage of apoptotic cells in thymi derived from HYRX-exposed mice (807.1 ± 78.87 vs. 1705 ± 194.5 cells/mm^3^, p < 0.0001, respectively) which was mostly localized in the thymic medulla and was significantly reduced by MEx treatment (865.5 ± 117.6 cells/mm^3^, p < 0.0001) (**Figures 6F, G**). These results demonstrate that MEx confer protective effects in medullary TECs and DCs.

### Single Cell RNA Sequencing Shows a Preferential Effect of MEx Treatment on the Transcriptome of Thymic Myeloid Cell Populations and mTECs

**Figure 7:**
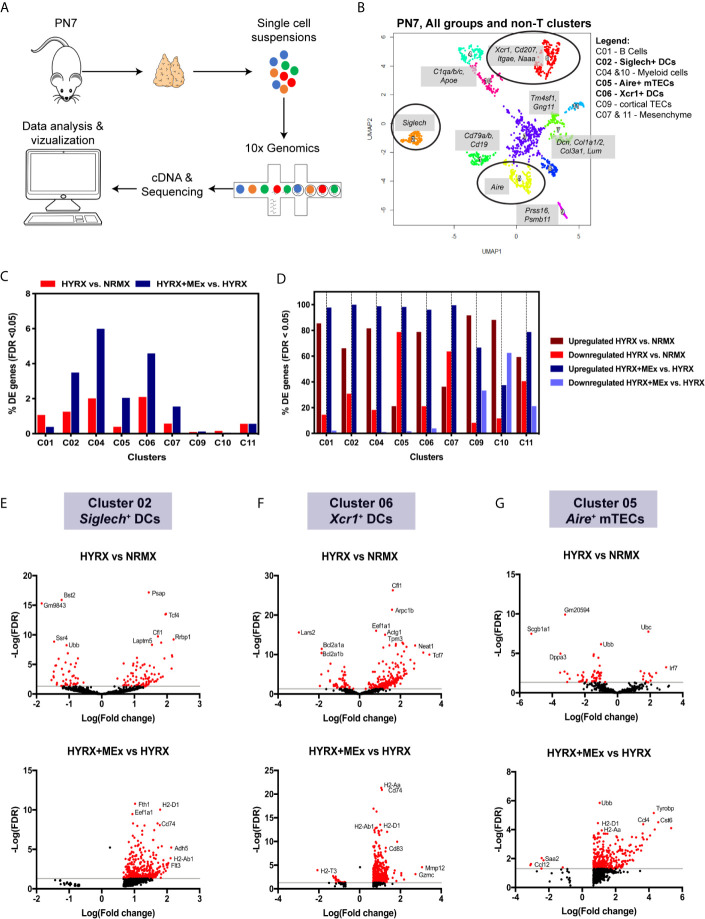
Single cell transcriptomics shows a preferential effect of MEx treatment on thymic DC populations and mTECs. **(A)** Schematics showing the experimental layout for single cell transcriptomics of thymi from NRMX, HYRX and HYRX+MEx groups. Newborn mice were exposed to HYRX for 7 days and MEx treatment performed at PN4 as previously described in the methods. Thymi (N = 3 per group, pooled) were harvested and prepared into single cell suspensions and cDNA libraries, sequencing and data analysis were performed as described in the methods section. **(B)** UMAP for the combined NRMX, HYRX and HYRX+MEx groups and all clusters, excluding T cells, were projected for PN7. Gene combinations that identify each numbered cluster are shown in grey boxes and black circles identify clusters 02, 06 and 05 corresponding to *Siglech^+^* DCs, *Xcr1^+^* DCs and *Aire^+^* mTECs. Legend shows which cell types are represented in each relevant cluster. **(C)** Percentage of differentially expressed (DE) genes showing a false discovery rate < 0.05 in relation to the total number of genes identified in non-T cell clusters in the pairwise comparisons HYRX vs NMRX (red) and HYRX+MEx vs HYRX (blue). **(D)** Percentage of upregulated and downregulated genes in all DE genes detected in non-T cell clusters. Dark red and light red identify percentage of upregulated and downregulated genes in HYRX vs NRMX group, and dark blue and light blue identify upregulated and downregulated genes in the comparison HYRX+MEx vs HYRX. Volcano plots showing differential gene expression differences for **(E)** cluster 02 **(F)** cluster 06 and **(G)** cluster 05 for the two relevant pair-wise comparisons (HYRX vs NRMX and HYRX+MEx vs NRMX) are shown. Genes highlighted in red exhibit an absolute log2(FC) > 1 and a false discovery rate (FDR) < 0.05.

To generate mechanistic leads on MEx induced modulation of thymic medulla, thymi from NRMX, HYRX and HYRX + MEx - treated mice (pooled N= 3 for each condition) were collected and processed into single cell suspensions for whole thymus single cell RNA transcriptomics using the 10x Genomics platform (**Figure 7A**). Numbers of sequenced cells, unique molecular identifiers (UMIs) and genes/cell for each condition are described in **Table S2**. A Uniform Manifold Approximation and Projection (UMAP) for the whole thymus of the three different conditions was generated and differential gene expression of the different populations was calculated (**Figure S7**and **Tables S3** and **S4**). MEx modulated gene expression differences were majorly detected in the non-T cell populations of the thymus at PN7. Whole thymus samples derived from NRMX, HYRX and HYRX+MEx on PN14 were also analyzed, but no major differences were detected (data not shown). On the basis of this data, a new UMAP containing only the non-T cell lineages, i.e., B, myeloid, epithelial and mesenchymal cells was generated (**Figure 7B**). Cluster 01 identified a population of *Cd79a/b* ^+^ and *Cd19* ^+^ B cells, while clusters 04 and 10 identified populations of myeloid cells characterized by the expression of *Apoe* and *C1qa/b/c.* Clusters 03 and 08 identified empty droplets and contaminants and were not included in further analysis. Cluster 09 identified *Prss16^+^Psmb11^+^* cortical TECs and clusters 07 and 11 represented *Dcn^+^* and *Tm4sf^+^Gng11^+^* mesenchymal cells. Clusters 02 and 06 represented *Siglech^+^* and *Xcr1^+^* DCs, respectively and cluster 05 identified *Aire^+^* mTECs. The highest percentage of differentially expressed genes (DEG), i.e., False Discovery Rate (FDR) < 0.05, was detected in the DC clusters 02 and 06, myeloid cell population in cluster 04, and mTECs in cluster 05 (**Figure 7C**). Analysis of the percentage of upregulated and downregulated genes in the two analyzed pair-wise comparisons of HYRX vs NRMX and HYRX+MEx vs HYRX showed that MEx promoted transcript upregulation in clusters of myeloid cells (e.g. DCs) and mTECs, with >90% of upregulated transcripts (**Figure 7D**). In view of data reported in this study on histological assessment of the DC and mTEC populations in the analyzed groups, further analysis was focused on the DC clusters 02, 06 and the mTEC cluster 05 (circled in UMAP shown in **Figure 7B**). Volcano plots in **Figures 7E–G** show the DEG distribution in HYRX vs NRMX and HYRX+MEx vs HYRX comparisons in *Siglech^+^* and *Xcr1* ^+^ DCs and *Aire* ^+^ mTECs. Compared with NRMX, there were 90 upregulated and 40 downregulated transcripts in HYRX- *Siglech^+^* DCs. MEx treatment induced a transcript upregulation, with 379 upregulated and 0 downregulated genes in HYRX+MEx compared to HYRX (**Figure 7E**, **Table S5**). Similarly, there were 209 upregulated and 56 downregulated genes in HYRX- *Xcr1^+^* DCs compared to NRMX, and 557 upregulated and only 23 were downregulated in the HYRX+MEx vs HYRX comparison (**Figure 7F**, **Table S4** **).** *Aire* ^+^ mTECs showed a similar trend in DEG distribution with 14 upregulated and 52 downregulated genes in HYRX vs NRMX compared to 339 upregulated and only 6 downregulated in HYRX+MEx vs HYRX comparison (**Figure 7F**, **Table S5** **)**. Volcano plots showing the DEG distribution in the other clusters are shown in **Figure S8** and **Table S5**. Genes showing the highest Log(Fold changes) and -Log(FDR) are labelled in each volcano plot for each comparison and cluster.

### MEx Treatment of HYRX-Exposed Mice Promoted the Upregulation of Transcripts Related to Antigen Presentation and Anti-Oxidation by Thymic DCs and mTECs

**Figure 8:**
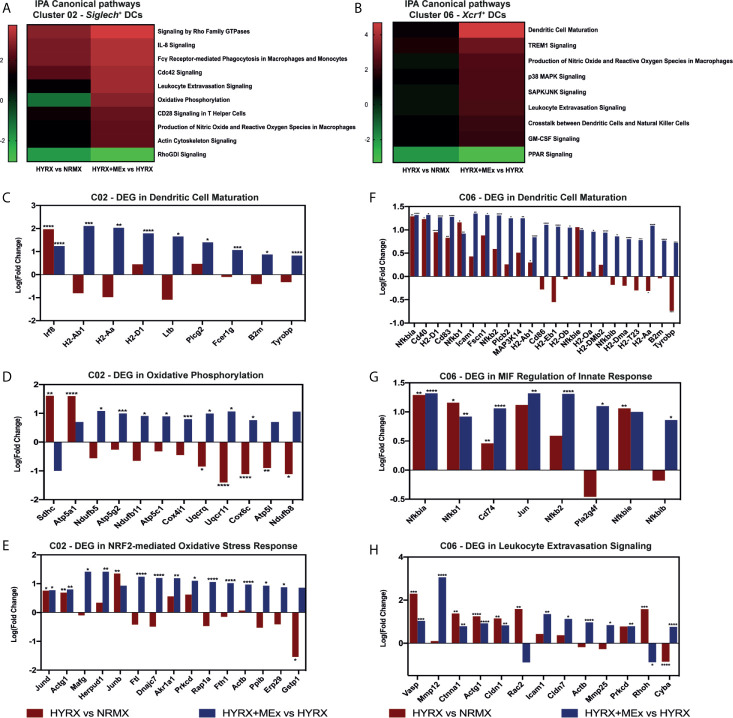
Canonical pathway analysis comparison for the two analyzed pair-wise comparisons for the *Siglech^+^* DC cluster 02 and *Xcr1^+^* DC cluster 06. Canonical pathway enrichment for clusters 02 and 06 for the comparisons HYRX vs NRMX and HYRX+MEx vs HYRX was performed using IPA as described in the methods section. **(A)** Heatmap showing activation Z scores for enriched pathways in Cluster 02. **(B)** Heatmap showing activation Z scores for enriched pathways in Cluster 06. Only pathways showing a Z score > 2 and <-2 and a fold change (HYRX+MEx vs MEX/HYRX vs NRMX) > 1.5 are shown. Using IPA software, genes involved in relevant activated pathways in MEx treated samples were queried and graphed. Fold changes showing the differentially expressed genes (DEGs) in HYRX vs NRMX (red) and HYRX+MEx vs HYRX (blue) comparisons for cluster 02 involved in the pathways **(C)** Dendritic Cell Maturation, **(D)** Oxidative Phosphorylation and **(E)** NRF2-mediated Oxidative Stress Response and for cluster 06 **(F)** Dendritic Cell Maturation, **(G)** MIF regulation of Innate Response and **(H)** Leukocyte Extravasation Signaling. Data derived from a pooled cohort of N = 3 per group and * P < 0.05, **P < 0.01, ***P < 0.001, ****P < 0.0001.

To generate a global overview of HYRX and MEx effects on DC clusters 02 and 06 and mTECs, ingenuity pathway analysis (IPA) was used to investigate and compare DEG in the pairwise comparisons HYRX vs NRMX and HYRX+MEx vs HYRX. The IPA comparison analysis of DEG of the DC clusters 02 and 06 showed an enrichment of pathways related to innate immune function and activation, which showed higher activation scores after MEx treatment (**Figures 8A, B**). Clusters 02 and 06 derived from MEx treated subjects exhibited an activation of additional pathways related to DC maturation, antigen presentation and migration, e.g., Dendritic Cell Maturation (Z score = 2.65) by cluster 02 and MIF Regulation of Innate Immunity (Z score = 2.83) by cluster 06 (**Table 1**). Compared to HYRX, clusters 02 and/or 06 derived from MEx treated samples presented upregulated profiles of genes related to co-stimulation and antigen presentation (e.g., histocompatibility antigen genes *H2-Ab1* and *H2-Aa*, *Cd83*, *Cd86*, Transmembrane immune signaling adaptor (*Tyrobp)*, among others) and leukocyte migration (e.g., intracellular adhesion molecule 1 (*Icam1)*, actin ß (*Actb)*, claudin 7 (*Cldn7)*, cytochrome B-245 alpha chain (*Cyba)*). In HYRX group these genes were either downregulated or showed no differences in gene expression compared to NRMX (**Figures 8C, F–H** **)**.

**Table 1:** Canonical pathways with an activation Z score detected only in the pair-wise comparison HYRX+MEx vs HYRX in Clusters 02 – *Siglech* ^+^ DCs and 06 – *Xcr1* ^+^ DCs.

Clusters	Canonical Pathways	Activation Z Score	
		HYRX vs NRMX	HYRX+MEx vsHYRX
**02 - *Siglech*^+^ DCs**	NRF2-mediated Oxidative Stress Response	N/A	3.16
	Role of NFAT in Regulation of the Immune Response	N/A	3.00
	CXCR4 Signaling	N/A	2.89
	Dendritic Cell Maturation	N/A	2.65
	mTOR Signaling	N/A	2.45
	Inhibition of ARE-Mediated mRNA Degradation Pathway	N/A	2.24
	BEX2 Signaling Pathway	N/A	2.00
	Th1 Pathway	N/A	2.00
**06 - *Xcr1*^+^ DCs**	MIF Regulation of Innate Immunity	N/A	2.83
	IL-1 Signaling	N/A	2.65
	TNFR2 Signaling	N/A	2.53
	TNFR1 Signaling	N/A	2.53
	iNOS Signaling	N/A	2.45
	Interferon Signaling	N/A	2.24
	LPS-stimulated MAPK Signaling	N/A	2.12
	Fatty Acid β-oxidation I	N/A	2.00

Interestingly, MEx derived cluster 02 cells presented with an enrichment of genes linked to oxidative stress response pathways, e.g., NRF2-mediated Oxidative Stress Response (Z score = 3.16, N/A in HYRX vs NRMX) and Oxidative Phosphorylation Pathway (Z score = 2.83 vs -1.13 in HYRX vs NRMX) (**Table 1** and **Figure 8A**). Compared to NRMX, HYRX-derived cluster 02 cells showed a downregulation of genes related to protection against oxidative stress responses e.g., ubiquinol-cytochrome c reductase complex III subunit XI (*Uqcr11)*, ATP synthase subunit g (*Atp5l)* and glutathione S-transferase P (*Gstp1)* which were upregulated by MEx treatment (**Figures 8D, E**).

**Figure 9:**
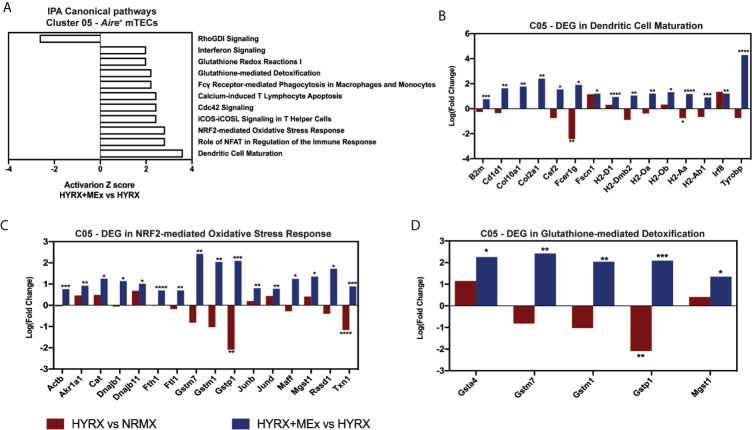
Canonical pathway analysis for the *Aire^+^* mTECs cluster 05. **(A)** graph showing canonical pathways activated or inhibited in HYRX+MEx vs HYRX comparison. There were no activation or inhibition Z scores detected in the HYRX vs NRMX comparison for these pathways as a result of low DEG number detected. Fold changes showing the DEGs in HYRX vs NRMX (red) and HYRX+MEx vs HYRX (blue) comparisons for cluster 05 involved in the pathways **(B)** Dendritic Cell Maturation, **(C)** NRF2-mediated Oxidative Stress Response and **(D)** Glutathione-mediated Detoxification. Data derived from a pooled cohort of N = 3 per group and * P < 0.05, **P < 0.01, ***P < 0.001, ****P < 0.0001.

Similar results were detected by MEx treatment in *Aire^+^* mTEC cluster. The DEG profile of MEx treated mTECs showed an activation of Dendritic Cell Maturation pathway with an upregulation of genes like Fc fragment of IgE receptor Ig (*Fcer1g)*, *H2-Aa* and *Tyrobp* compared to HYRX (**Figures 9A, B**). These cells also presented with an activation score of pathways related to response to oxidative stress (NRF2-mediated Oxidative Stress Response and Glutathione-mediated Detoxification) (**Figure 9A**). Compared to NRMX, HYRX-derived mTECs presented with downregulated profiles of oxidative stress response genes *Gstp1* and *Txn1*, which were upregulated along with other relevant oxidant stress response genes upon MEx treatment (**Figures 9C, D**). These results indicate that MEx confers an anti-oxidative effect to thymic DC and mTEC populations thus preventing their senescence and promoting their differentiation into mature antigen presenting cells which will drive thymocyte selection and maturation.

## Discussion

Although the effects of neonatal hyperoxia exposure on the thymic development of naïve T cell pools are not yet known, some studies indicate that, coupled with a disruption of thymic architecture, irreversible alterations to the T cell compartment occur (11–13). BPD, the better studied chronic lung disorder that often occurs in preterm newborns requiring oxygen supplementation and/or mechanical ventilation, manifests as a systemic disease with multiorgan effects in addition to lung injury associated with inflammation. MEx based therapies have been shown to modulate the immune system, significantly suppressing inflammation in the hyperoxic lung and promoting amelioration of the core features of BPD (23–25) and are now being considered as a promising therapeutic approach for that disease. However, further assessment is necessary to unravel the mechanism of action and multiorgan effects of MEx treatment during neonatal hyperoxia, and especially its modulatory effects on HYRX-induced disruption of thymic morphology and T cell development.

In this study we demonstrate that a single dose of MEx administered during neonatal HYRX exposure improved thymic medullary architecture, restored thymocyte counts, regulatory T cell numbers, promoted thymocyte differentiation into CD4^+^ T cells and reduced oxygen-induced T cell autoreactivity *in vitro*. Furthermore, using immunofluorescence, TUNEL and single cell RNA sequencing, we extended on current knowledge on the effect of HYRX on thymic medullary cells. HYRX-exposed mice presented loss of Aire expression by mTECs and loss of CD11c^+^ DC numbers and increased apoptosis levels which were mostly concentrated in the medullary regions. Notably, we are the first to demonstrate that MEx treatment during HYRX-exposure restored Aire^+^ mTECs and CD11c^+^ DC numbers and concomitantly blunted apoptosis levels in the medullary region of treated thymi. Additionally, we demonstrate by single-cell transcriptomics that MEx have a targeted effect on thymic myeloid cells, and more specifically on DCs and mTECs which showed enrichment of transcripts involved in maturation/antigen presentation and in antioxidant defense pathways.

The beneficial effects of MEx have been demonstrated in various preclinical investigations. Studies published by our group unraveled mechanistic insights on MEx immunomodulation in models of inflammation-driven lung disorders. We demonstrated that MEx blunted inflammation, either tissue-specific or systemic, *via* the modulation of myeloid cell populations (23, 26). HYRX-exposure during the neonatal period has been shown to have phenotypic and functional repercussions on the thymus and T cell compartment. Using a baboon model, Rosen et al. demonstrated an oxygen-induced disruption of thymic architecture which resulted in the release of autoreactive T cells and immunodeficiency (11). Two other independent preclinical studies revealed that HYRX-exposed thymi presented with increased apoptosis rates and irreversible changes in T cell phenotypes which persisted into adulthood (12, 13). Our results are in accordance with published data by showing drastic reduction of thymic medullary areas in HYRX-exposed mice, loss of thymocytes and increased apoptosis. Furthermore, we are the first to show that MEx treatment restored these parameters to healthy control levels.

MEx capacity to modulate immune cell functions or activities has been demonstrated in various studies (33–36). MEx were reported to promote a T helper phenotype by converting Th1 to Th2 and reducing Th17 differentiation in peripheral blood mononuclear cells (PBMCs) (37, 38). More importantly, MEx increased the levels of FoxP3^+^ regulatory T cells in PBMCs *via* the suppression of pro-inflammatory cytokines, e.g. TNFα and IL-1ß and increase in anti-inflammatory cytokine expression, e.g., TGFß (35). In fact, MEx surface has been shown to be decorated by anti-inflammatory TGFß-1 (39, 40). Data reported in our study show a recovery of thymic FoxP3^+^ expression within the CD4^+^ thymocytes with a concomitant increase of regulatory T cells frequencies in spleen and lungs. Interestingly, at this level of analysis and timepoints examined, we did not detect significant differences in T cell profiles and activation phenotypes in the peripheral organs, such as in lungs and spleen. In such a dynamic environment whereby developing neonatal T cells are undergoing maturation and populating peripheral organs, these results are likely a reflection of T cell profiles at the analyzed timepoints. Further investigations are warranted to fully resolve how HYRX and MEx-treatment may impact the developing peripheral T cell phenotypes over time. Additionally, we show that MEx promoted the differentiation of the thymocytes into a CD4^+^ helper phenotype in detriment of a CD8^+^ cytotoxic phenotype, shifting CD4/CD8 ratios into a T helper phenotype. It is important to note that results presented herein are reflective of whole CD4^+^ and CD8^+^ phenotypes. In the mouse, it is well established that the developing double negative thymocytes undergo CD8 upregulation which gives rise to an immature CD8^+^ thymocyte population (41). In addition, MEx-treated mice also presented with increased frequencies of a known intermediate thymocyte phenotype characterized as CD4^+^CD8^low^. As previously reported, this population has the capacity to differentiate into either SP8 or SP4 thymocytes (32). Therefore, whether this MEx-driven differentiation into a CD4 phenotype is long-lasting in the thymus needs to be investigated in further studies. Nonetheless, it is fair to speculate that one of the mechanisms of MEx regulation of the differentiation of T cells into a CD4 and regulatory phenotype is *via* the delivery of anti-inflammatory components encased by or displayed on their bilipid membrane.

The main function of the thymic medulla is to negatively select the developing thymocytes for the generation of mature naïve T cells. After migrating to the thymus, the developing thymocyte progenitors undergo a succession of selection processes modulated by a complex 3D network of stromal and non-stromal cells, of which thymic epithelial cells and dendritic cells are of utmost importance. In the thymic cortex, the early progenitors, still lacking the expression of the known T cell markers CD4 and CD8 (DN population), interact with cortical TECs for positive selection. This process gives rise to DP thymocytes that may express TCRs with high affinity to self-antigens (5, 6). Interestingly, in our study we detected no HYRX-induced changes in cortical thymocytes, with similar DN and DP frequencies detected across the groups, including in the MEx-treated thymi. Changes in thymocyte phenotypes and functions were detected at the later stages of differentiation (SP4/8 populations), which is controlled by the thymic medulla. In this compartment, an interaction between medullary TECs and DCs drives the presentation of self-antigens to the developing thymocytes. A disruption or a loss of this interaction has been shown to lead to increased T cell autoreactivity, loss of regulatory T cells and autoimmunity (42–45). During the neonatal period, these mechanisms are of paramount importance since it is during this period that a population of regulatory T cells migrates to tissue and maintains central tolerance throughout life (46, 47). HYRX-exposure resulted in loss of thymic medulla which in turn hampered negative selection mechanisms, including the elimination of autoreactive T cells. Our data are in accordance with the study published by Rosen et al. which showed increased T cell proliferation when these cells were primed with autologous lung cells (11). More importantly, autoreactivity of splenic and thymic T cells to autologous lung cells was reduced to control levels in MEx-treated animals. The data described herein could be interpreted to reflect changes in both autoreactive T cells as well as regulatory T cells in HYRX-exposed mice. Whether this increased HYRX-induced T cell autoreactivity is a direct result of evasion of autoreactive T cells from negative selection or a decrease in regulatory T cell generation, remains to be fully elucidated. Our data indicate that MEx-driven blunting of T cell autoreactivity may be in part due to increased regulatory T cell populations at both thymic and splenic levels. Given the importance of medullary TECs and DCs in controlling the selection of these cells, we focused our attention on this cellular compartment.

The interplay between TECs and DCs plays important roles in thymocyte maturation and the development of thymic medulla (48). Published data have shown that HYRX-induced T cell modifications were in part due to the disruption of thymic ‘nurse cells’ (11), which refer to the population of cortical TECs driving positive selection of thymocytes. Contrary to what was first shown by Rosen et al. (11), our data showed disruption of the thymic medulla and, consequently, medullary TECs upon HYRX-exposure, instead of thymic ‘nurse cells’. Medullary TECs represent a special type of cell that, when mature, express Aire, the transcription factor which will allow for the expression and presentation of a plethora of self-antigens. Current knowledge has demonstrated that the maturation of these cells into Aire^+^ mTECs largely depends on thymic and recruited CD11c^+^ DCs (49–51). We found that neonatal HYRX-exposure led to a loss of CD11c^+^ DCs, a decreased number of Aire^+^ mTECs, and increased apoptosis. It is important to note that although increased apoptosis in the HYRX-exposed mice was detected mainly in the thymic medullary area, with the technique utilized in this study, it is not possible to distinguish between cell types underdoing apoptosis in the thymic medulla. Likely, apoptotic cells represent both the medullary stroma and a big proportion of thymocytes in response to the increased oxygen levels. More importantly, MEx-treated thymi manifested an increase of CD11c^+^ DCs and Aire expression by mTECs and normalized medullary apoptosis levels.

We postulated that the regulatory mechanisms of MEx on these cells could reflect the changes detected in T cells. We employed single-cell RNA transcriptomics analysis to investigate mechanistic insights of MEx on the various thymic cell populations. In this study, we demonstrated that thymic myeloid cells and mTECs were the cell types mostly modulated by MEx treatment. Of the myeloid cell clusters identified, *Siglech* DCs in cluster 02 and *Xcr1* DCs in cluster 06 showed high percentages of DEGs with most of the transcripts showing an upregulated profile. The expression of *Siglech* has been associated with a population of precursor-DCs which are recruited to the thymus from the bone marrow. Once in the thymus, these pre-DCs mature into an antigen presenting phenotype, interact with other antigen presenting cells and mTECs and present self-antigens to drive thymocyte selection (52). *Xcr1* DCs represent a type of antigen presenting cells whose migration to the thymus is controlled by an XCL1 gradient produced by *Aire^+^* mTECs (49). Interestingly, our single cell transcriptomics showed that, upon MEx treatment, the DEGs in the *Siglech* DC cluster 02 were all upregulated compared to HYRX. Canonical pathway analysis comparison further revealed that, compared to HYRX, both DC clusters expressed higher levels of genes that play important roles in antigen presentation pathways and dendritic cell maturation. Moreover, *Aire^+^* mTECs showed a similar pattern of gene enrichment and canonical pathway activation scores, indicating a mature and functional phenotype which in the thymus, is essential for thymocyte selection and development.

Furthermore, both MEx-treated DCs and mTECs collectively showed an upregulation of genes that are pivotal in NRF2-mediated Oxidative Stress Response, Oxidative Phosphorylation and Glutathione-mediated Detoxification, which represent the main cellular defense mechanisms against oxidative stress (53–55). Growing evidence suggests that the thymic antigen presenting cell axis is susceptible to oxidative stress-induced senescence. Animal studies of chemically induced oxidative stress in the thymus linked the increased oxidant status to thymic involution (9, 10). Additionally, investigations in thymic ageing demonstrated that irreversible thymic atrophy results from the incapacity of the thymic stroma to eliminate hydrogen peroxide (H_2_O_2_), a major by-product of oxidative stress thus leading to increased tissue damage (8). Notably, administration of antioxidative compounds have been shown to significantly decrease thymic oxidative damage and restore thymic morphology (10, 56). In our study, we demonstrate a MEx-modulated upregulation of genes such as, *Atp5l*, NADH : Ubiquionone Oxireductase Subunit 8 (*Ndufb8*) and Cytochrome C Oxidase Subunit 6C (*Cox6c*), in DC cluster 02, which have known roles in the mitochondrial respiratory chain and antioxidant defense mechanisms (55, 57). Moreover, MEx-modulated mTECs were shown to upregulate genes such as glutathione S-transferase mu 7 (*Gstm7)*, glutathione S-transferase mu 1 (*Gstm1)* and glutathione S-transferase alpha 4 (*Gsta4)*, which are part of the glutathione S transferase (GST) family of enzymes that catalyzes compounds for detoxification (54). Interestingly, both MEx-modulated DC clusters and mTECs exhibited increased expression of *Gstp1* gene. This gene encodes an enzyme that has a critical role in decreasing oxidative damage in cells by scavenging the reactive oxygen species (ROS) to glutathione which then eliminates these toxic compounds (58). Effects of EVs on ROS scavenging and detoxification has been previously demonstrated by various groups, and these vesicles have been shown to either carry different antioxidant enzymes such as glutathione peroxidase (GPX), glutathione S-transferase (GST) or catalase (CAT) (59–61) or to contain the necessary enzymatic machinery to activate antioxidant pathways (62). Additionally, in a mouse model of hepatic oxidant injury, human umbilical cord derived MEx have been shown to prevent ROS formation in hepatocytes *via* the delivery of GPX1 (63). Altogether, these results indicate that MEx induce a protective effect on the thymic antigen presenting cell axis, i.e., DCs and mTECs, by promoting the activation of anti-oxidative pathways during HYRX exposure, allowing for efficient maturation and function of these cells and inhibiting morphological damage of the thymic medulla.

Our results showed a preferential effect of MEx on thymic DCs and mTECs. Notably, the identified thymic DCs are of peripheral origin and migrate to the thymus for antigen presentation to the developing thymocytes. It is plausible to postulate that migratory DCs are targeted by MEx in the bloodstream and propagate MEx effects to other cell types in the thymus. *In vivo* biodistribution assays using MEx labelled with a near infrared dye and *in vivo* imaging performed by our group have not detected significant MEx accumulation in the thymus (data not shown), however, due to limitations with currently used biodistribution technology, we cannot fully exclude direct homing of MEx to the thymus or uptake by cell types other than the migratory DCs. Future studies should focus on deciphering the mechanisms of action of MEx by in-depth study of their components and subtypes. This may represent a rather daunting task, since the beneficial effects of MEx are probably the result of a well-orchestrated synergy by a plethora of bioactive components rather than the action of a single molecular entity. In the context of our model and the observed restoration of thymic morphology and function, we note that our experimental approach to administer MEx at PN4 during hyperoxia exposure, ensures that these vesicles are present during an essential period for perinatal development of T cells (46, 47). Whether MEx would sustain the same effect in the thymus if given at different timepoints should be elucidated in further studies. Nonetheless, ours is the first study showing that intravenous administration of MEx can protect the thymus from oxygen-induced damage of the thymic medulla during hyperoxia exposure typically occurring in preterm infants in the perinatal period, thus protecting it from injury and cellular loss while promoting the normal development of thymic T cells. Important T cell populations that maintain central tolerance and protect against foreign pathogens throughout life are developed during the perinatal period. A disruption of these mechanisms by neonatal hyperoxia-induced dysregulation of thymic T cell development may cause long lasting defects in this compartment and lead to the development of autoimmune disorders and/or immunodeficiency. Most importantly, MEx induced protection of thymic morphology and function during neonatal-hyperoxia exposure represents a promising therapeutic avenue to prevent the later onset of autoimmune and other disorders resulting from a disruption of the normal neonatal T cell compartment.

## Data Availability Statement

The datasets presented in this study can be found in online repositories. The names of the repository/repositories and accession number(s) can be found below: NCBI (https://www.ncbi.nlm.nih.gov/sra/PRJNA715227).

## Ethics Statement

The animal study was reviewed and approved by Boston Children’s Hospital Animal Care and Use Committee.

## Author Contributions

MR participated in study design and execution, data collection, analysis and manuscript writing. GW participated in study design and execution and data collection. AF-G participated in study design and execution and data collection. VY participated in study execution and data collection. ET participated to study execution and data collection. MM participated in data collection. TP participated in data collection. AD participated in data analysis. RM provided infrastructure and advice in support of single cell RNA-sequencing experiments. SAM and SK contributed to study design, supervision of study execution, manuscript writing, data analysis, and article editing and approval. All authors contributed to the article and approved the submitted version.

## Funding

This work was supported in part by NIH grants R01HL146128 and R21AI134025 (SK); United Therapeutics Research Grant (SK and SAM); American Thoracic Society Foundation Grant and NIH K99HL146986 (GW); and Charles H. Hood Foundation Major Grants Initiative to Advance Child Health (SK). EST was supported by NIH T32HD098061 Neonatal Research Training Program.

## Conflict of Interest

SK and SAM are named inventors on intellectual property licensed by Boston Children’s Hospital to United Therapeutics Corp.

The remaining authors declare that the research was conducted in the absence of any commercial or financial relationships that could be construed as a potential conflict of interest.

## Supplementary Material

Supplementary Figure 1 Gating strategy for identification of the different thymocyte phenotypes. Single cell suspensions were stained with anti-mouse CD44, CD25, CD4, CD8 and FoxP3 antibodies and analysed by flow cytometry for the different thymocyte populations. **(A)** Live cells were selected according to their side scatter (SSC-A) and forward scatter (FSC-A) properties and single cells gated by according to their SSC-A/SSC-H and FSC-A/FSC-H characteristics. Selected cells were plotted against CD8 and CD4 for the selection of double negative (DN, CD4^-^CD8^-^), double positive (DP, CD4^+^CD8^+^, single positive 4 (SP4, CD4^+^CD8^-^) and single positive 8 (SP8, CD4^-^CD8^+^) thymocytes. The different DN stages was assessed by gating the DN population and analysing the expression of CD44 and CD25, and, DN1 = CD44^+^CD25^-^, DN2 = CD44^+^CD25^+^, DN3 = CD44^-^CD25^+^ and DN4 = CD44^-^CD25^-^. **(B)** Gating of thymic regulatory T cells was performed by gating CD25^+^FoxP3^+^ population within the SP4 population.

Supplementary Figure 2 Gating strategy for identification of lung and spleen regulatory T cells. Single cell suspensions of PN14 lungs and spleens were stained with anti-CD45, CD3, CD4, CD8, CD25 and FoxP3 antibodies for phenotyping of regulatory T cells. **(A)** Lung cells and **(B)** spleen cells were selected according to SSC-A and FSC-A properties. Single cells were gated by plotting SSC-A and FSC-A against SSC-H and FSC-H, respectively. The cell gate was further analysed for CD45 and CD3 expression, and CD4 T cells were discriminated by plotting CD4 versus CD8 and gating CD4^+^CD8^-^ population. **(C)** Regulatory T cells were gated by selection of the CD4^+^ and plotting CD25 versus FoxP3. Regulatory T cell phenotype was determined as CD4^+^CD25^+^FoxP3^+^ T cells. Blue counter plot represents fluorescence minus one control after which gates were drawn and red counter plot shows an example of FoxP3 expression. **(D)** Gating strategy for CFSE dilution studies used for assessment of T cell autoreactivity. Live cells were gated based on SSC-A and FSC-A properties. Single cells were gated by plotting SSC-A against SSC-H and T cells were then gated based on CD4 or CD8 expression. Thymocytes were gated based on CD4 and CD8 expression while splenocytes were first selected by CD3 expression. Dilution od CFSE was then gated based on unstimulated CFSE-labelled CD4 or CD8 cells.

Supplementary Figure 3 Characterization of WJ-MSCs. Passage 3 WJ-MSCs were assessed for their morphology, differentiation capacity and phenotype. **(A)** WJ-MSCs exhibited the characteristic spindle shaped morphology. Representative micrographs of osteogenic and adipogenic differentiation of WJ-MSCs show calcium deposition (visualized using Alizarin Red S staining), and lipid droplet formation (detected by Oil Red O staining), respectively, after culture in the presence of commercially available differentiation media. **(B)** Representative flow cytometry histograms show that WJ-MSCs are positive for the expression of the surface markers CD73, CD90, CD105, CD44 and CD146 and negative for the expression of lineage specific markers, CD34, CD19, CD11b, CD14 and HLA-DR.

Supplementary Figure 4 Immunoblotting films of MEx characterization. Representative western blot films of fractions 1-6 and 7-12 for the assessment of exosomal proteins in MEx preparations used in this work. MEx was assessed for the expression of the exosomal proteins CD63, CD81, SDCBP and TSG101 and the non-exosomal protein Calnexin was used as a purity marker.

Supplementary Figure 5 Thymocyte phenotypes at PN7. **(A)** Representative flow cytometry graphs of DN1-4 phenotypes based on the expression of CD44 and CD25 (see **Figure S1A** for gating strategy) for NRMX, HYRX and MEx treated groups. Frequency of **(B)** DN1, **(C)** DN2, **(D)** DN3, and **(E)** DN4 thymocytes in the three different experimental groups. **(F)** Representative flow cytometry graphs of DN, DP, SP4 and SP8 populations (gated as described in **Figure S4A**) in NRMX, HYRX and HYRX + MEx groups. Frequency of **(G)** DN, **(H)** DP, **(I)** SP4 and **(J)** SP8 populations in the three analysed groups. Data represent mean ± SEM of N = 8 of at least two independent experiments.

Supplementary Figure 6 Analysis of DN phenotypes at PN14. **(A)** representative and pooled frequencies of the **(B)** DN1, **(C)** DN2, **(D)** DN3 and **(E)** DN4 populations in single cell suspensions of thymi harvested from NRMX, HYRX and MEx treated groups as assessed by the expression of CD44 and CD25 and gated as described in **Figure S1A**. Data represent mean ± SEM of N = 8-9 of at least two independent experiments.

Supplementary Figure 7 UMAP projections of thymic single cell RNA sequencing. Thymi from NRMX, HYRX and HYRX+MEx groups (N = 3 for each group), were prepared into single cell suspensions, pooled and sequenced using 10x Genomics. UMAPs for all cell clusters and all groups were projected as described in the methods section for PN7. Gene combinations that identify each cluster are shown in proximity to the clusters in the grey boxes.

Supplementary Figure 8 Volcano plots showing differential gene expression differences for non-T cell clusters. **(A)** cluster 01 – B cells, **(B)** cluster 04 – myeloid cells, **(C)** cluster 10 – other myeloid cells, **(D)** cluster 09 – cortical TECs, **(E)** cluster 07 and **(F)** cluster 11 representing mesenchymal cells. Volcano plots for the two relevant pair-wise comparisons (HYRX vs NRMX and HYRX+MEx vs NRMX) are shown. Genes highlighted in red exhibit an absolute log2(FC) > 1 and a false discovery rate (FDR) < 0.05.

Supplementary Table 1 List of reagents used in 10x Genomics single cell RNA sequencing experiments. Summary of extra kits and reagents, suppliers and product numbers used for single cell RNA sequencing experiments.

Supplementary Table 2 Quality control for each obtained transcriptome using the 10x Genomics single cell sequencing platform. The table shows the number of cells, unique molecular identifiers (UMIs) and genes/cell in each sample after data filtering.

Supplementary Table 3 Genes differentially expressed (DE) between pair-wise group combinations HYRX vs NRMX and HYRX+MEx vs HYRX for each T cell cluster identified in PN7. Tables show Log2 (Fold Changes [FC]) and false discovery rate (FDR). Genes that displayed a Log2(FC) > 1 and <-1 and FDR <0.05 are considered statistically significant. UMAP for clusters herein identified are shown in **Figure S8**.

Supplementary Table 4 Genes differentially expressed between the pair-wise group combinations HYRX vs NRMX and HYRX+MEx vs HYRX for each non-T cell cluster identified in PN7. Tables show Log2 (Fold Changes [FC]) and false discovery rate (FDR). Genes that displayed a Log2(FC) > 1 and <-1 and FDR <0.05 are considered statistically significant. UMAP displaying these clusters are shown in **Figure 7b**.

Supplementary Table 5 Number of upregulated and downregulated genes in non-T cell clusters. Table shows the number of DEGs in the two relevant pair-wise combinations, HYRX vs NRMX and HYRX+MEx vs NRMX.
