# Supplementary figures and images for: Mesenchymal Stromal Cell-Derived Extracellular Vesicles Restore Thymic Architecture and T Cell Function Disrupted by Neonatal Hyperoxia
Source: Front Immunol. 2021 Apr 15;12:640595. doi: 10.3389/fimmu.2021.640595 (PMC8082426; doi:10.3389/fimmu.2021.640595)

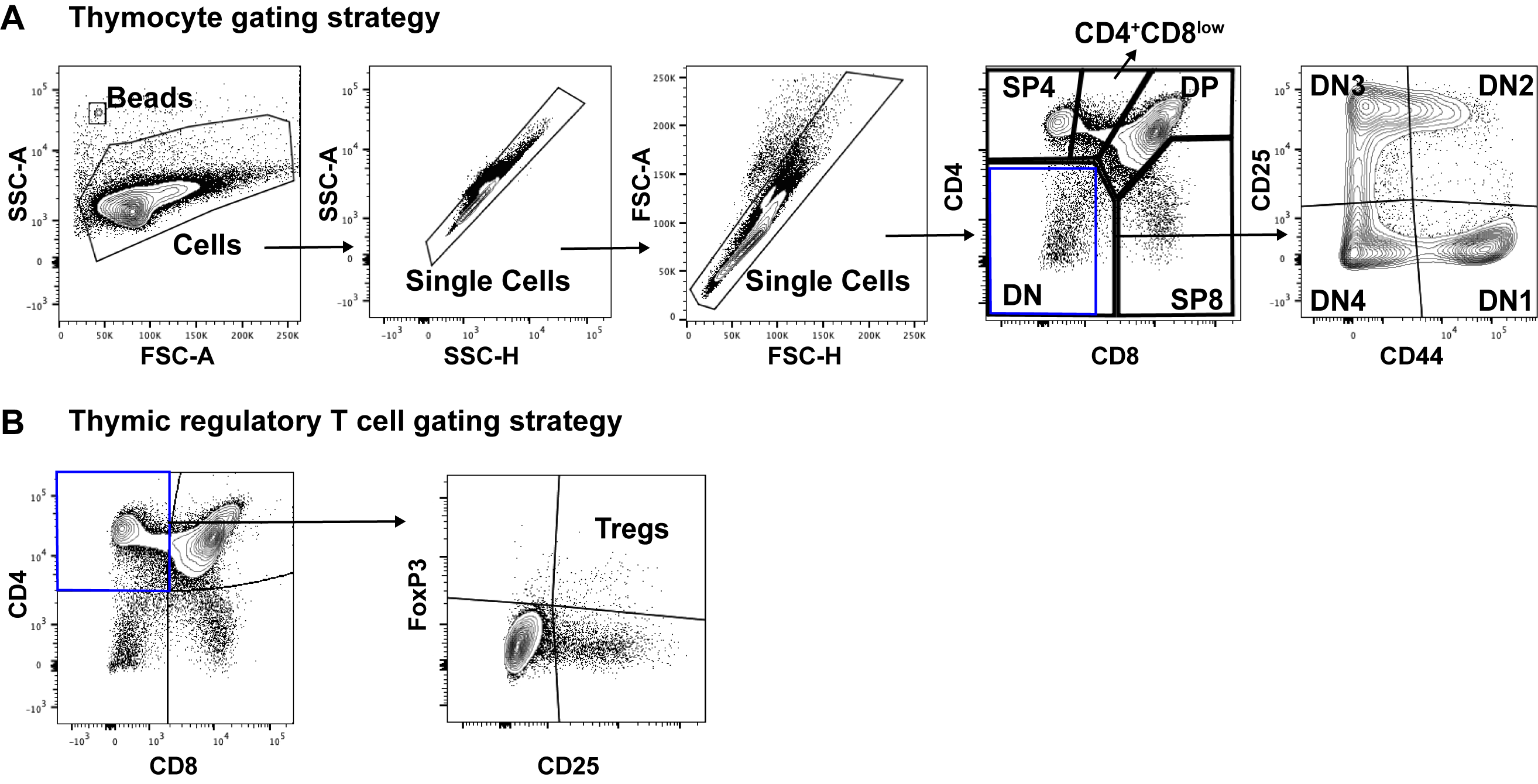

Supplement: Supplementary Figure 1 — Gating strategy for identification of the different thymocyte phenotypes. Single cell suspensions were stained with anti-mouse CD44, CD25, CD4, CD8 and FoxP3 antibodies and analysed by flow cytometry for the different thymocyte populations. (A) Live cells were selected according to their side scatter (SSC-A) and forward scatter (FSC-A) properties and single cells gated by according to their SSC-A/SSC-H and FSC-A/FSC-H characteristics. Selected cells were plotted against CD8 and CD4 for the selection of double negative (DN, CD4-CD8-), double positive (DP, CD4+CD8+, single positive 4 (SP4, CD4+CD8-) and single positive 8 (SP8, CD4-CD8+) thymocytes. The different DN stages was assessed by gating the DN population and analysing the expression of CD44 and CD25, and, DN1 = CD44+CD25-, DN2 = CD44+CD25+, DN3 = CD44-CD25+ and DN4 = CD44-CD25-. (B) Gating of thymic regulatory T cells was performed by gating CD25+FoxP3+ population within the SP4 population. [file Image_1.tif]

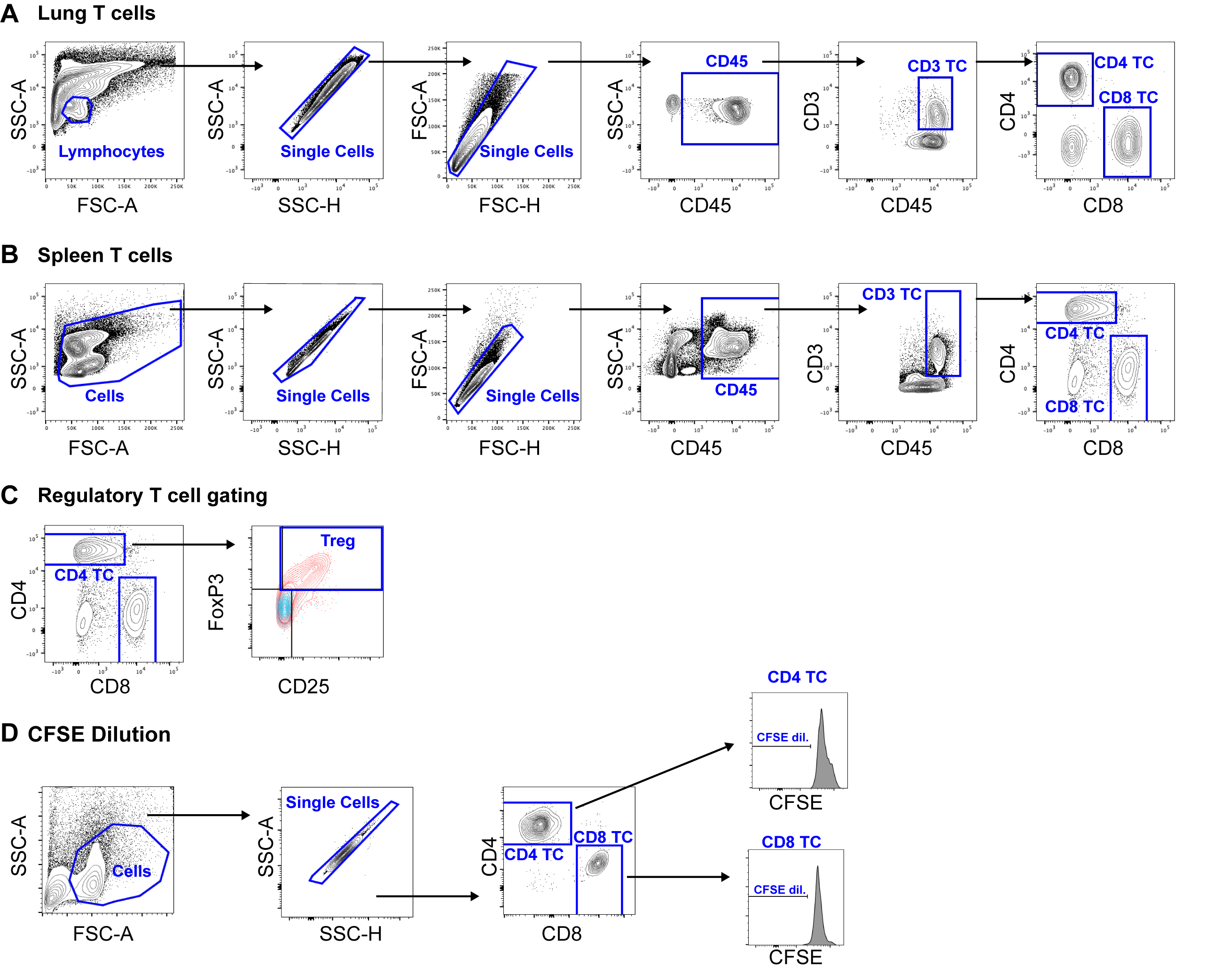

Supplement: Supplementary Figure 2 — Gating strategy for identification of lung and spleen regulatory T cells. Single cell suspensions of PN14 lungs and spleens were stained with anti-CD45, CD3, CD4, CD8, CD25 and FoxP3 antibodies for phenotyping of regulatory T cells. (A) Lung cells and (B) spleen cells were selected according to SSC-A and FSC-A properties. Single cells were gated by plotting SSC-A and FSC-A against SSC-H and FSC-H, respectively. The cell gate was further analysed for CD45 and CD3 expression, and CD4 T cells were discriminated by plotting CD4 versus CD8 and gating CD4+CD8- population. (C) Regulatory T cells were gated by selection of the CD4+ and plotting CD25 versus FoxP3. Regulatory T cell phenotype was determined as CD4+CD25+FoxP3+ T cells. Blue counter plot represents fluorescence minus one control after which gates were drawn and red counter plot shows an example of FoxP3 expression. (D) Gating strategy for CFSE dilution studies used for assessment of T cell autoreactivity. Live cells were gated based on SSC-A and FSC-A properties. Single cells were gated by plotting SSC-A against SSC-H and T cells were then gated based on CD4 or CD8 expression. Thymocytes were gated based on CD4 and CD8 expression while splenocytes were first selected by CD3 expression. Dilution od CFSE was then gated based on unstimulated CFSE-labelled CD4 or CD8 cells. [file Image_2.tif]

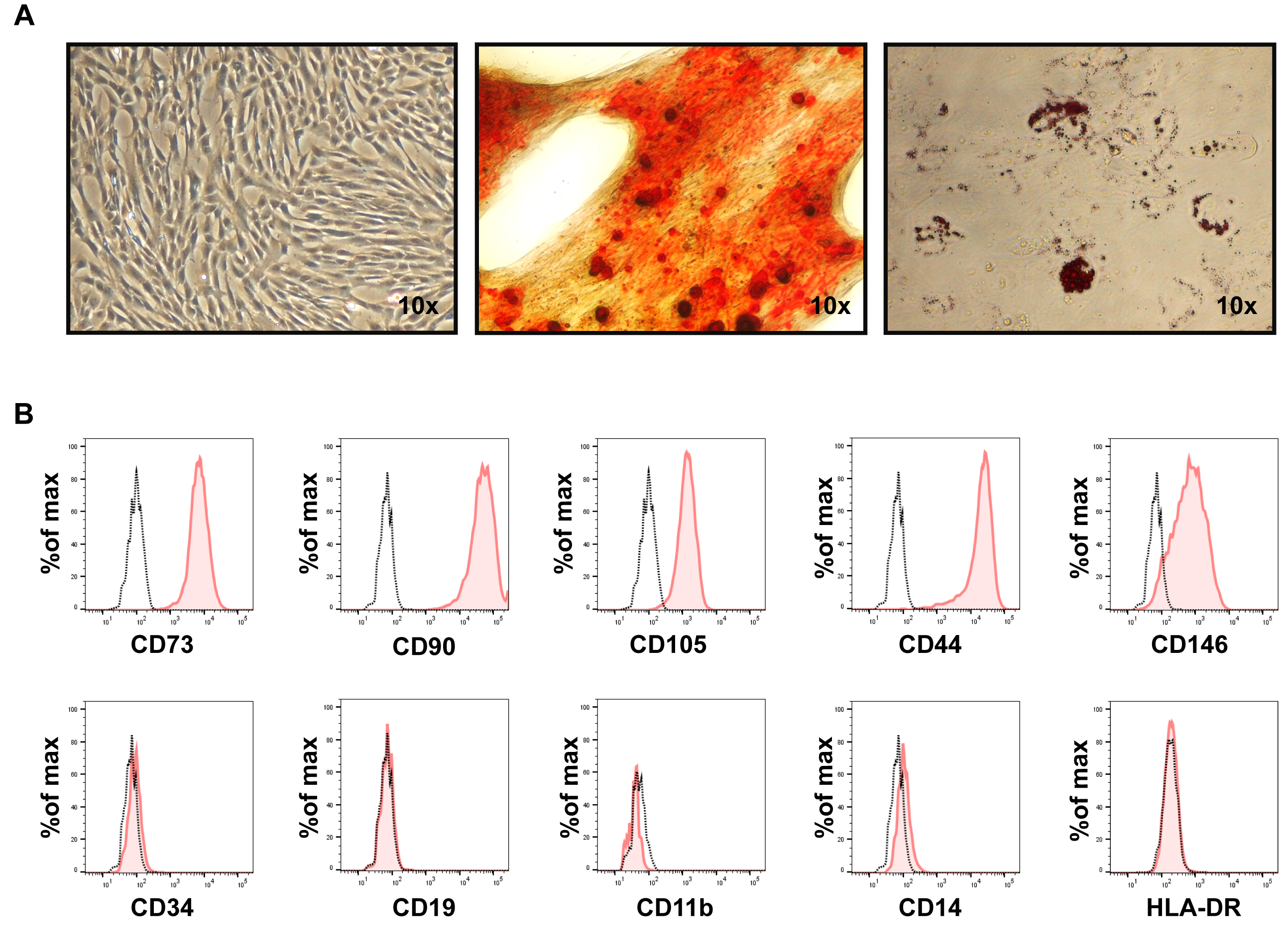

Supplement: Supplementary Figure 3 — Characterization of WJ-MSCs. Passage 3 WJ-MSCs were assessed for their morphology, differentiation capacity and phenotype. (A) WJ-MSCs exhibited the characteristic spindle shaped morphology. Representative micrographs of osteogenic and adipogenic differentiation of WJ-MSCs show calcium deposition (visualized using Alizarin Red S staining), and lipid droplet formation (detected by Oil Red O staining), respectively, after culture in the presence of commercially available differentiation media. (B) Representative flow cytometry histograms show that WJ-MSCs are positive for the expression of the surface markers CD73, CD90, CD105, CD44 and CD146 and negative for the expression of lineage specific markers, CD34, CD19, CD11b, CD14 and HLA-DR. [file Image_3.tif]

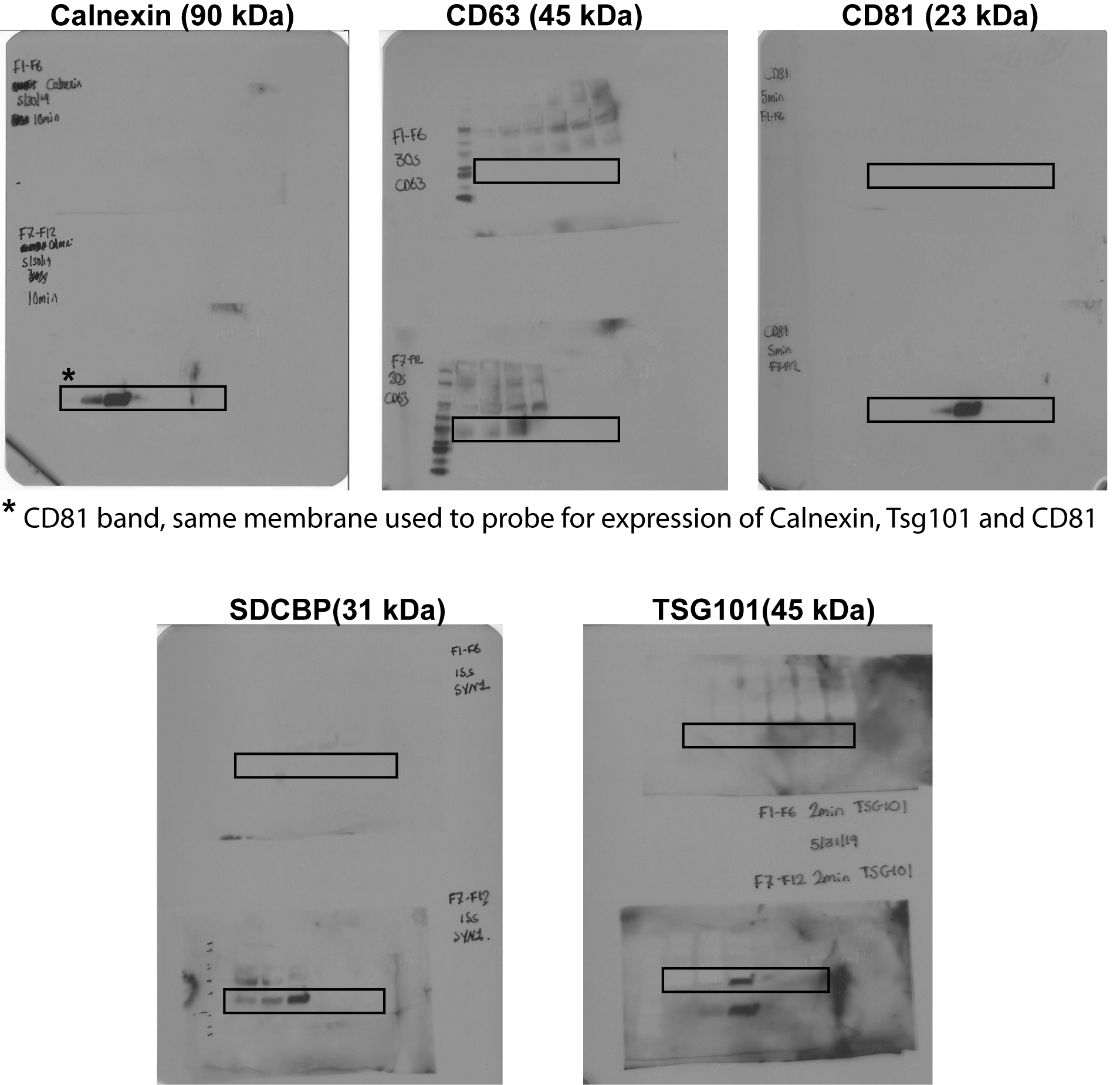

Supplement: Supplementary Figure 4 — Immunoblotting films of MEx characterization. Representative western blot films of fractions 1-6 and 7-12 for the assessment of exosomal proteins in MEx preparations used in this work. MEx was assessed for the expression of the exosomal proteins CD63, CD81, SDCBP and TSG101 and the non-exosomal protein Calnexin was used as a purity marker. [file Image_4.tif]

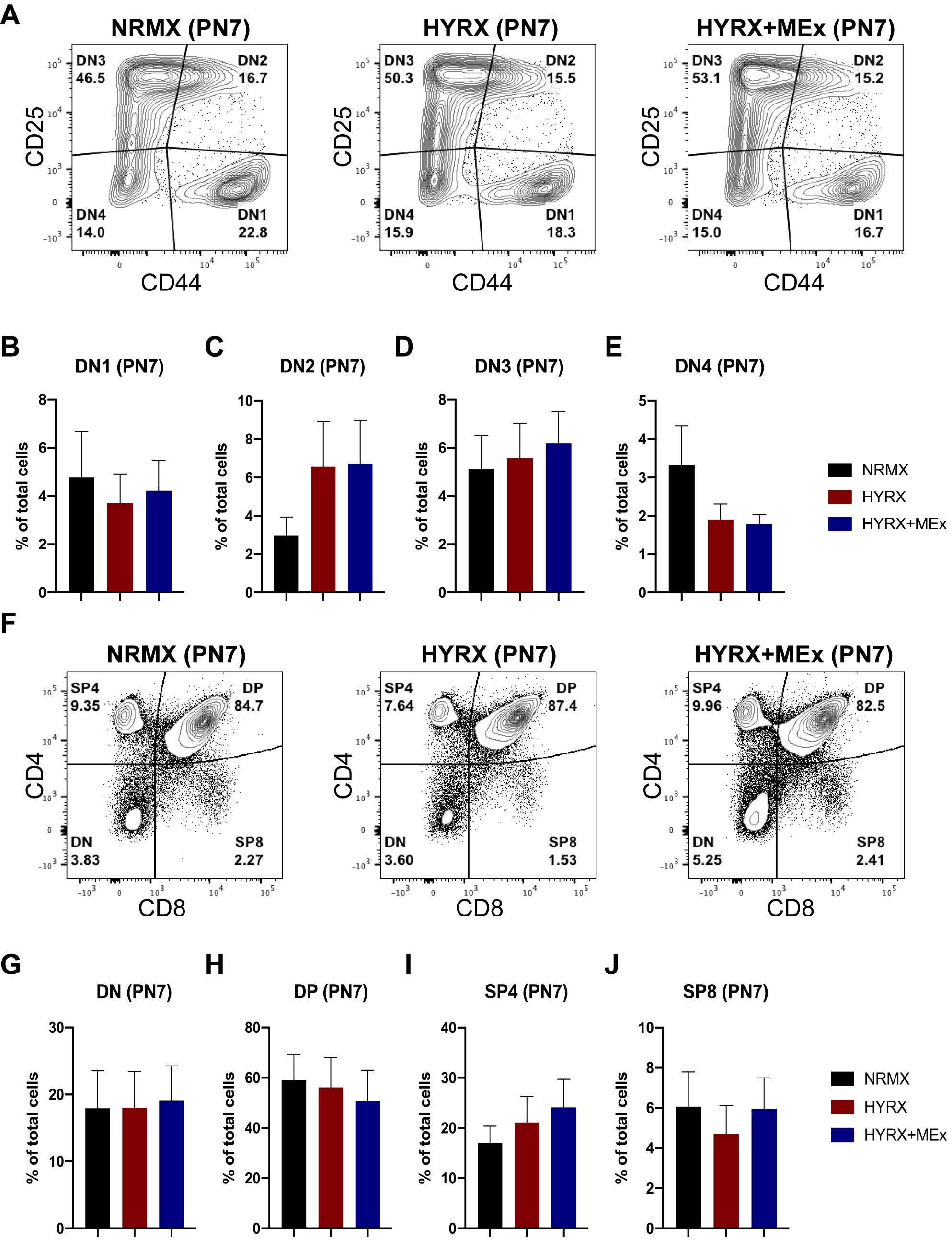

Supplement: Supplementary Figure 5 — Thymocyte phenotypes at PN7. (A) Representative flow cytometry graphs of DN1-4 phenotypes based on the expression of CD44 and CD25 (see Figure S1A for gating strategy) for NRMX, HYRX and MEx treated groups. Frequency of (B) DN1, (C) DN2, (D) DN3, and (E) DN4 thymocytes in the three different experimental groups. (F) Representative flow cytometry graphs of DN, DP, SP4 and SP8 populations (gated as described in Figure S4A ) in NRMX, HYRX and HYRX + MEx groups. Frequency of (G) DN, (H) DP, (I) SP4 and (J) SP8 populations in the three analysed groups. Data represent mean ± SEM of N = 8 of at least two independent experiments. [file Image_5.tif]

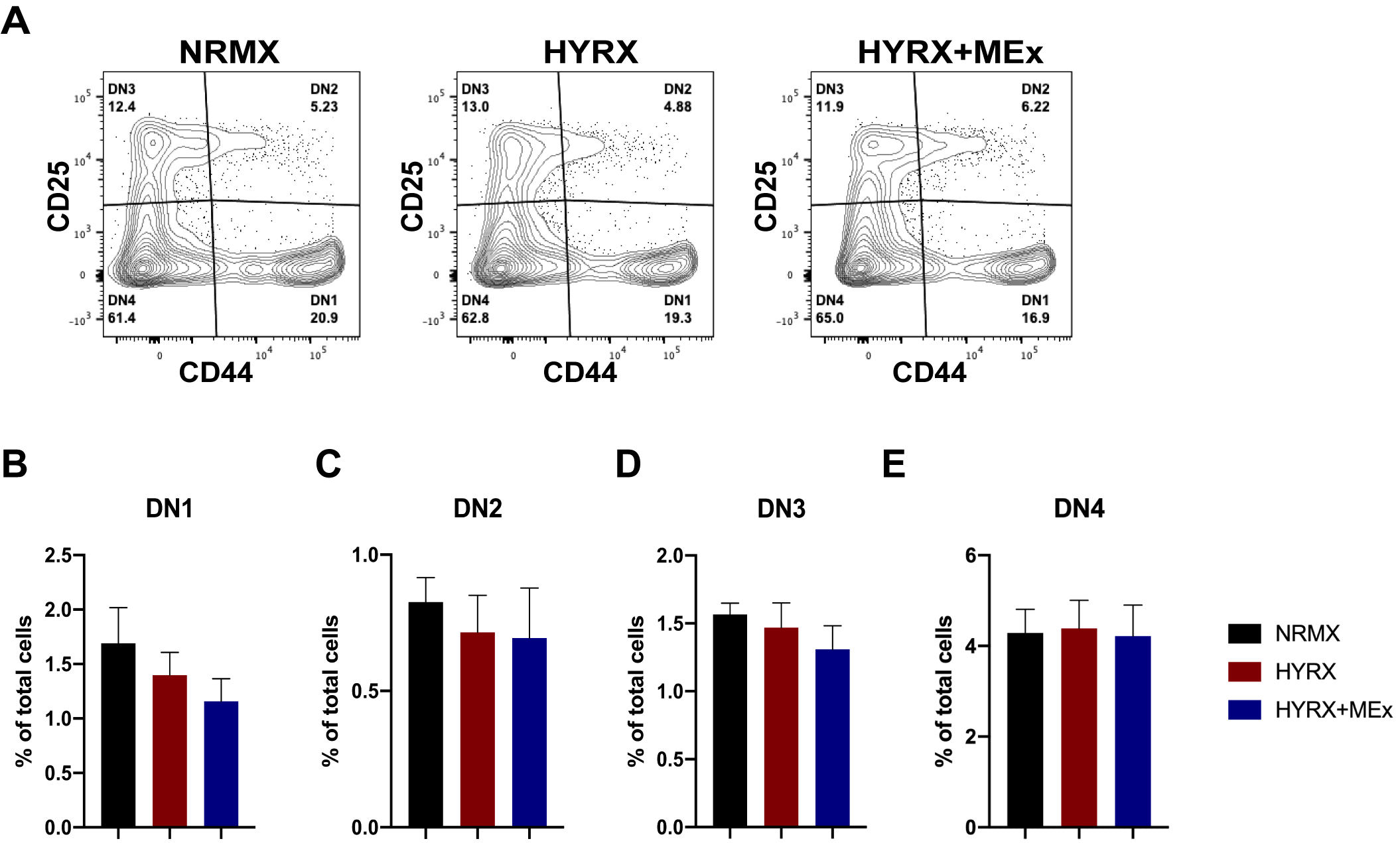

Supplement: Supplementary Figure 6 — Analysis of DN phenotypes at PN14. (A) representative and pooled frequencies of the (B) DN1, (C) DN2, (D) DN3 and (E) DN4 populations in single cell suspensions of thymi harvested from NRMX, HYRX and MEx treated groups as assessed by the expression of CD44 and CD25 and gated as described in Figure S1A . Data represent mean ± SEM of N = 8-9 of at least two independent experiments. [file Image_6.tif]

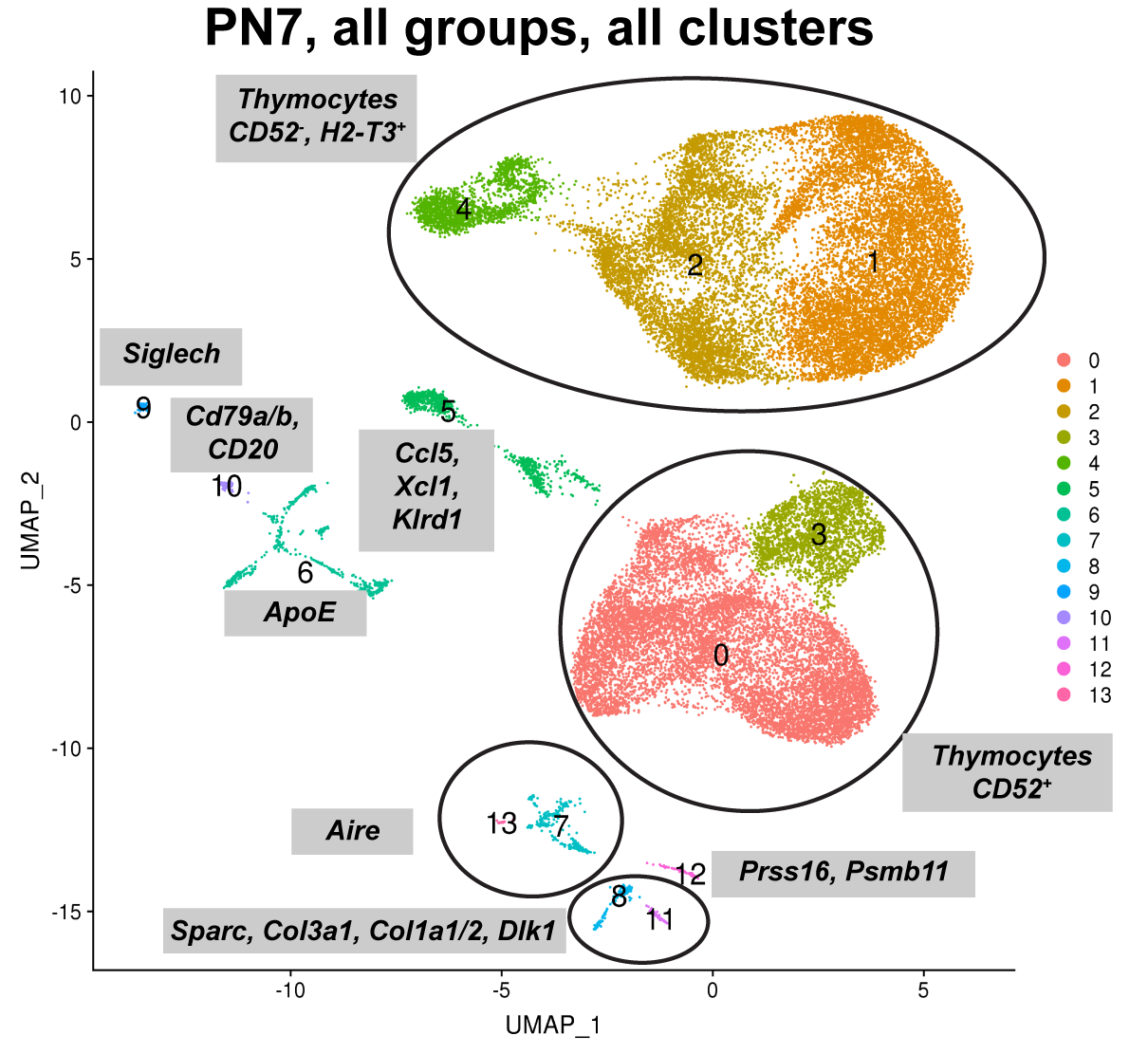

Supplement: Supplementary Figure 7 — UMAP projections of thymic single cell RNA sequencing. Thymi from NRMX, HYRX and HYRX+MEx groups (N = 3 for each group), were prepared into single cell suspensions, pooled and sequenced using 10x Genomics. UMAPs for all cell clusters and all groups were projected as described in the methods section for PN7. Gene combinations that identify each cluster are shown in proximity to the clusters in the grey boxes. [file Image_7.tif]

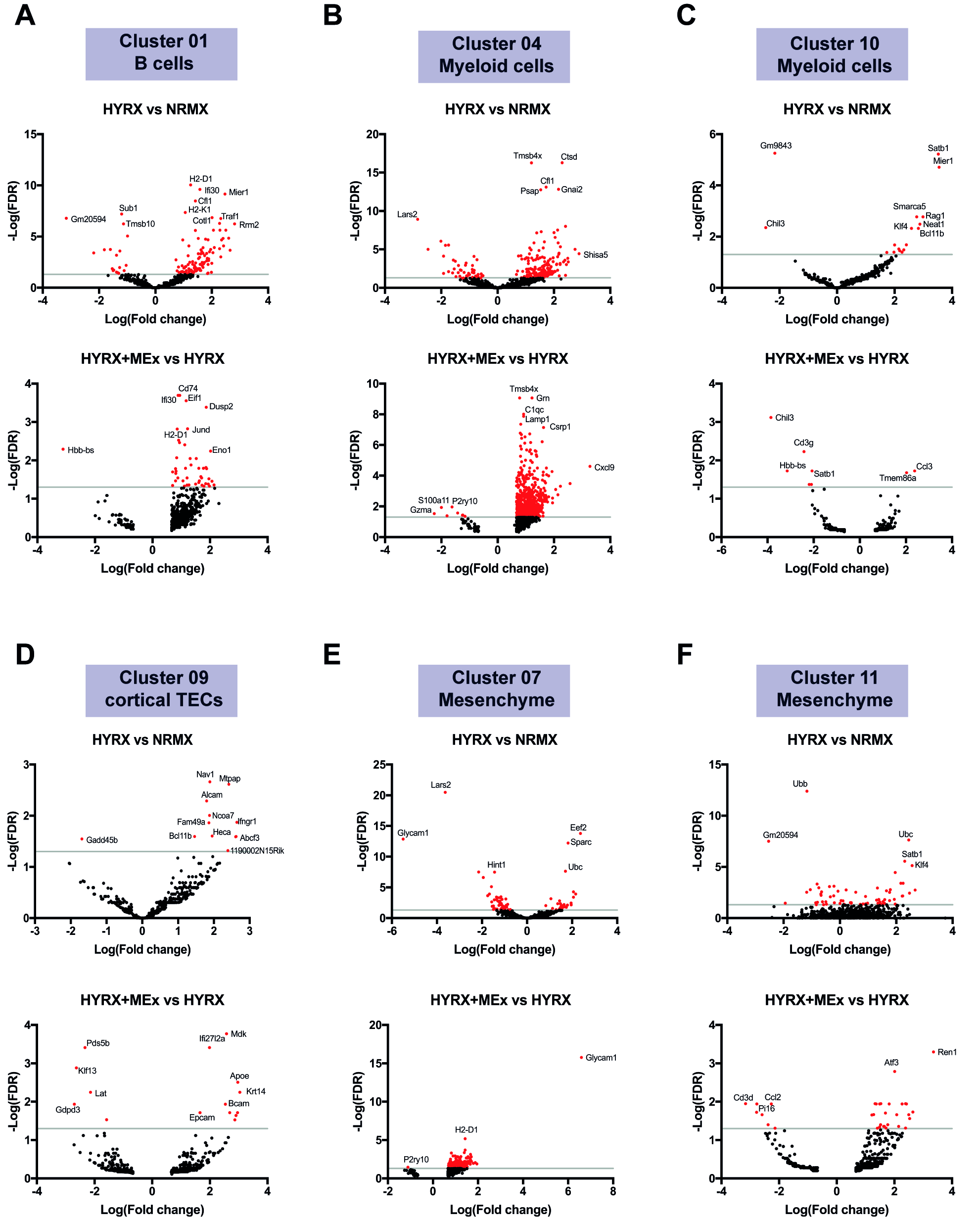

Supplement: Supplementary Figure 8 — Volcano plots showing differential gene expression differences for non-T cell clusters. (A) cluster 01 – B cells, (B) cluster 04 – myeloid cells, (C) cluster 10 – other myeloid cells, (D) cluster 09 – cortical TECs, (E) cluster 07 and (F) cluster 11 representing mesenchymal cells. Volcano plots for the two relevant pair-wise comparisons (HYRX vs NRMX and HYRX+MEx vs NRMX) are shown. Genes highlighted in red exhibit an absolute log2(FC) > 1 and a false discovery rate (FDR) < 0.05. [file Image_8.tif]
