# Supplementary material for: Mesenchymal Stromal Cell-Derived Extracellular Vesicles Restore Thymic Architecture and T Cell Function Disrupted by Neonatal Hyperoxia
Source: Front Immunol. 2021 Apr 15;12:640595. doi: 10.3389/fimmu.2021.640595 (PMC8082426; doi:10.3389/fimmu.2021.640595)
Supplement: Supplementary Table 1 — List of reagents used in 10x Genomics single cell RNA sequencing experiments. Summary of extra kits and reagents, suppliers and product numbers used for single cell RNA sequencing experiments. [file DataSheet_1.docx]

**Mesenchymal stromal cell-derived extracellular vesicles restore thymic architecture and T cell function disrupted by neonatal hyperoxia**

Monica Reis^1,2^, Gareth R. Willis^1,2^, Angeles Fernandez-Gonzalez^1,2^, Vincent Yeung^1,2^, Elizabeth Taglauer^1,2^, Margaret Magaletta^3^, Teagan Parsons^3^, Alan Derr^3^, Xianlan Liu^1,2^, Rene Maehr^3^, Stella Kourembanas^1,2^, and S. Alex Mitsialis^1,2^

Affiliations:

^1^Division of Newborn Medicine & Department of Pediatrics, Boston Children’s Hospital, Boston, Massachusetts, USA

^2^Department of Pediatrics, Harvard Medical School, Boston, Massachusetts, USA

^3^Program in Molecular Medicine, Diabetes Center of Excellence, University of Massachusetts Medical School, Worcester, MA, USA

Correspondence and requests for reprints should be addressed to: Dr. Stella Kourembanas, Boston Children’s Hospital, 300 Longwood Avenue, Division of Newborn Medicine, Boston, MA 02115

Tel: 617-919-2355. Fax: 617-730-0260. E-mail: stella.kourembanas@childrens.harvard.edu

**Supplementary Tables:**

**Supplementary Table S1: List of reagents used in 10x Genomics single cell RNA sequencing experiments.** Summary of extra kits and reagents, suppliers and product numbers used for single cell RNA sequencing experiments.

| **Kits & Extra Reagents** | **Supplier** | **Product Number** |
| --- | --- | --- |
| **ChromiumTM Single Cell 3' GEM, Library & Gel Bead Kit v3, 4 rxns** | 10x Genomics | 1000092 |
| **ChromiumTM i7 Multiplex kit, 96 rxns** | 10x Genomics | 120262 |
| **ChromiumTM Chip B Single Cell Kit, 16 rxns** | 10x Genomics | 1000074 |
| **Nuclease-free Water** | Thermo Fisher Scientific | AM9937 |
| **Low TE Buffer (10mM Tris-HCl, pH = 8.0, 0.1 mM EDTA)** | Thermo Fisher Scientific | 12090-015 |
| **Ethanol, Pure (200 Proof, anhydrous)** | Sigma | E7023-500mL |
| **SPRIselect Reagent Kit** | Beckman Coulter | B23318 |
| **Glycerin (glycerol), 50% (v/v) Aqueous Solution** | Ricca Chemical Company | 3290-32 |
| **Qiagen Buffer EB** | Qiagen | 19086 |
| **Agilent High Sensitivity DNA bioanalyzer Chip** | Agilent | NA |
| **NextSeq 500/550 Hi Output Kit V2.5 75-cycle** | Illumina | 20024906 |

**Supplementary Table S2: Quality control for each obtained transcriptome using the 10x Genomics single cell sequencing platform.** The table shows the number of cells, unique molecular identifiers (UMIs) and genes/cell in each sample after data filtering.

|  | | **Total Cells** | **Unique Molecular Identifiers (UMIs)** | **Genes/cell** |
| --- | --- | --- | --- | --- |
| **PN7** | **NRMX** | 9430 | 3316 | 1060.6 |
|  | **HYRX** | 16364 | 3572.1 | 1585.5 |
|  | **HYRX+MEx** | 6728 | 7430.9 | 2416.4 |

**Supplementary Tables S3: Genes differentially expressed (DE) between pair-wise group combinations HYRX vs NRMX and HYRX+MEx vs HYRX for each T cell cluster identified in PN7.** Tables show Log2 (Fold Changes [FC]) and false discovery rate (FDR). Genes that displayed a Log2(FC) > 1 and <-1 and FDR <0.05 are considered significant. UMAP for clusters herein identified are shown in **Fig. S8**.

**Supplementary Tables S4: Genes differentially expressed between the pair-wise group combinations HYRX vs NRMX and HYRX+MEx vs HYRX for each non-T cell cluster identified in PN7.** Tables show Log2 (Fold Changes [FC]) and false discovery rate (FDR). Genes that displayed a Log2(FC) > 1 and <-1 and FDR <0.05 are considered significant. UMAP displaying these clusters are shown in **Fig. 7b**.

**Supplementary Table S5: Number of upregulated and downregulated genes in non-T cell clusters.** Table shows the number of DEGs in the two relevant pair-wise combinations, HYRX vs NRMX and HYRX+MEx vs NRMX.

|  |  | **Comparisons** | **C01** | **C02** | **C04** | **C06** | **C05** | **C07** | **C09** | **C10** | **C11** |
| --- | --- | --- | --- | --- | --- | --- | --- | --- | --- | --- | --- |
| **#Genes** | **Total genes** |  | 11601 | 10855 | 12460 | 12647 | 16880 | 13456 | 12243 | 10883 | 11335 |
|  | **Upregulated genes** | **HYRX vs NRMX** | 106 | 90 | 205 | 209 | 14 | 28 | 11 | 15 | 38 |
|  |  | **HYRX+MEx vs HYRX** | 44 | 379 | 738 | 557 | 339 | 208 | 10 | 3 | 26 |
|  | **Downregulated genes** | **HYRX vs NRMX** | 18 | 42 | 46 | 56 | 52 | 49 | 1 | 2 | 26 |
|  |  | **HYRX+MEx vs HYRX** | 1 | 0 | 9 | 23 | 6 | 1 | 5 | 5 | 7 |
